# Machine learning-driven exosome-mimetic lipid nanoparticles for tumor-specific targeting

**DOI:** 10.1186/s40580-026-00531-7

**Published:** 2026-01-28

**Authors:** Seongmin Ha, Do Hyun Lee, Taehoon Lee, Hairi Jiang, Hyun-jin Lee, Seungbum Seo, Ji Yeong Yang, Sunyoung Park, Sung-Gyu Park, Joonchul Shin, Hyo-Il Jung

**Affiliations:** 1 School of Mechanical Engineering, Yonsei University, 50 Yonsei-ro, Seodaemun-gu, Seoul, 120-749 Republic of Korea; 2 The DABOM Inc, 50 Yonsei-ro, Seodaemun-gu, Seoul, 120-749 Republic of Korea; 3 Department of Biomedical Technology, Kangwon National University, Chuncheon, Republic of Korea; 4 Advanced Bio and Healthcare Materials Research Division, Korea Institute of Materials Science (KIMS), Changwon, Gyeongnam Republic of Korea; 5 Department of Integrated Medicine, Yonsei University, 50 Yonsei-ro, Seodaemun-gu, Seoul, 120-749 Republic of Korea

**Keywords:** Exosome-mimetic lipid nanoparticles (ENPs), Machine-learning hybrid algorithm, Cancer-targeted nanomedicine, Critical material attributes (CMAs), Critical quality attributes (CQAs)

## Abstract

Exosome-mimetic lipid nanoparticles (ENPs) are a promising alternative to PEGylated lipid nanoparticles (LNPs) for targeted cancer therapy, offering improved biocompatibility and reduced immune clearance. However, the rational design of these biomimetic particles is challenging due to complex lipid composition requirements. We developed a hybrid algorithm to optimize exosome-mimetic formulations by predicting key nanoparticle properties (size, zeta potential, and polydispersity index). The algorithm was trained on an expanded dataset of 17,800 lipid compositions generated by augmenting experimental and publicly available data using the LipidGAN generative model, incorporating physicochemical modeling and feature extraction. It identified optimal formulations, which were validated in vitro across three cancer cell lines (HeLa, H1975, and MCF-7). Cytotoxicity assays confirmed minimal toxicity (cell viability > 90%), and uptake studies demonstrated efficient, cell-type-specific internalization (91 ~ 95%). These results highlight the potential of artificial intelligence (AI)-driven lipid design to emulate the functionality of natural exosomes and advance the development of safe, effective, and personalized cancer nanomedicines.

**Graphical abstract:**

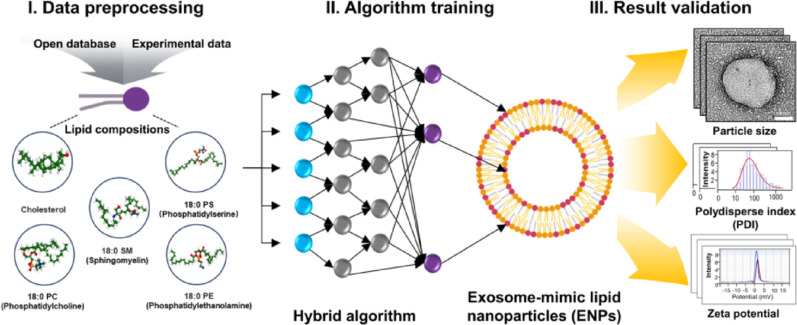

**Supplementary Information:**

The online version contains supplementary material available at 10.1186/s40580-026-00531-7.

## Introduction

Anticancer drugs mostly used in chemotherapy non-specifically attack tumors and normal cells, resulting in severe side effects [1]. These side effects substantially lower patient quality of life and limit therapeutic dosages. There is a growing interest to develop targeted drug delivery systems targeting tumor tissues while minimizing harm to normal, healthy tissues. Over the past few decades, researchers have introduced drug delivery integrated with nanotechnology to boost treatment effectiveness and reduce side effects [2–5]. Lipid nanoparticles (LNPs) have become promising carriers that effectively encapsulate drugs and deliver them directly to specific tissues. For instance, Doxil and Abraxane illustrate the promising potential of lipid nanoparticles (LNPs). Additionally, the successful integration of LNP technology in mRNA vaccines further emphasizes their significance as advanced drug delivery systems [6–9].

However, LNPs are prone to being rapidly recognized and cleared from the body by various biological defense mechanisms [10]. Several studies employed LNP surface modification with polyethylene glycol (PEG) to address this issue. This coating reduces immune recognition and extends the nanoparticles’ circulation time in the bloodstream [11]. PEGylated lipids generate a hydration layer around the nanoparticle surface, significantly decreasing protein attachment and clearance by macrophages. Consequently, nanoparticles remain in the bloodstream longer, particularly after their initial administration [12]. Also, PEGylated LNPs cause accelerated blood clearance (ABC). The production of anti-PEG antibodies prompts the immune system to eliminate the nanoparticles rapidly [13]. Note that recent studies have identified rare cases of anaphylactic shock in a small subset of patients following immunization with mRNA vaccines formulated with PEG-containing LNPs. Such findings have raised important safety considerations regarding the clinical use of PEG-based LNPs [14].

In this regard, multiple studies have demonstrated that anionic LNPs can encapsulate diverse therapeutics beyond nucleic acids. Ambati et al. achieved high encapsulation efficiencies (70 ~ 90%) for various proteins using anionic lipid formulations [15]. Likewise, Gabizon et al. co-encapsulated multiple chemotherapeutics (e.g., doxorubicin and a lipidated mitomycin-C) in PEGylated liposomes, improving combination therapy efficacy [16]. Additionally, Fatima et al. employed transmembrane pH gradients to load weakly basic drugs into liposomes with near-quantitative efficiency, eliminating the cationic lipids [17]. These findings support the use of anionic, exosome-mimetic LNPs (ENPs) over cationic formulations, avoiding associated toxicity while maintaining broad cargo.

Exosomes are nanoscale extracellular vesicles that mediate intercellular communication and have drawn considerable interest for applications in drug delivery, regenerative medicine, and diagnostics [18–20]. These natural vesicles offer inherent biocompatibility and the ability to carry diverse biomolecular cargo [21]. Natural exosomes are mostly composed of cholesterol (CHOL) and sphingomyelin (SM), along with phosphatidylcholine (PC), phosphatidylethanolamine (PE), and phosphatidylserine (PS) [22]. These compositions make the membrane more rigid and stable than cellular membranes [22]. Concerning these, exosomes can circulate in blood without rapid degradation [23]. These structural advantages contribute to the superior delivery efficiency and reduced immunogenicity of exosomes compared to LNPs [24]. Still, identifying an optimal lipid formulation that confers the desired stability, cargo capacity, and biological activity is a non-trivial task, given the enormous design space of possible lipid combinations [25]. To address these issues, artificial intelligence (AI)-driven ENP design has recently gained attention [26, 27].

AI technology has emerged as an effective tool for rational ENP design. Conventional trial-and-error approaches to formulating ENP are inefficient due to the enormous combinatorial complexity and limited understanding of how specific lipid mixtures influence nanoparticle stability and therapeutic efficacy [28]. It overcomes these challenges by efficiently analyzing high-dimensional formulation data to identify optimal lipid compositions rapidly. Recent studies demonstrate that the machine learning (ML) model accelerates formulation development significantly, uncovers hidden nonlinear relationships between lipid composition and nanoparticle performance and enables precise tailoring of ENP properties for specific therapeutic targets [29, 30]. However, this data-driven approach faces challenges such as limited scalability and restricted generalization due to inherent biases in training datasets. Our study incorporates a dataset from experimental results and an open database, significantly enhancing the clinical translational potential of ML-powered natural exosome-mimic designs.

Herein, we present a hybrid ML-based algorithm to navigate the high-dimensional formulation space of ENPs efficiently. We designed an artificial intelligence model to identify optimal lipid composition ratios predicted to enhance uptake in three cancer cell lines: HeLa (cervical cancer), H1975 (lung cancer), and MCF-7 (breast cancer). The designed ENPs were composed exclusively of five naturally occurring lipids - CHOL, SM, PC, PE, and PS - intentionally excluding synthetic PEG-lipids and cationic lipids to improve biomimicry and enhance biocompatibility. The term ‘exosome-mimetic’ refers strictly to the emulation of the solely lipid compositions found in natural exosomes. The ML-driven lipid compositions were subsequently used to formulate ENPs and in-vitro evaluation: cellular uptake efficiency, cytotoxicity, and target specificity to identify the most effective ENP formulations.

**Fig. 1:**
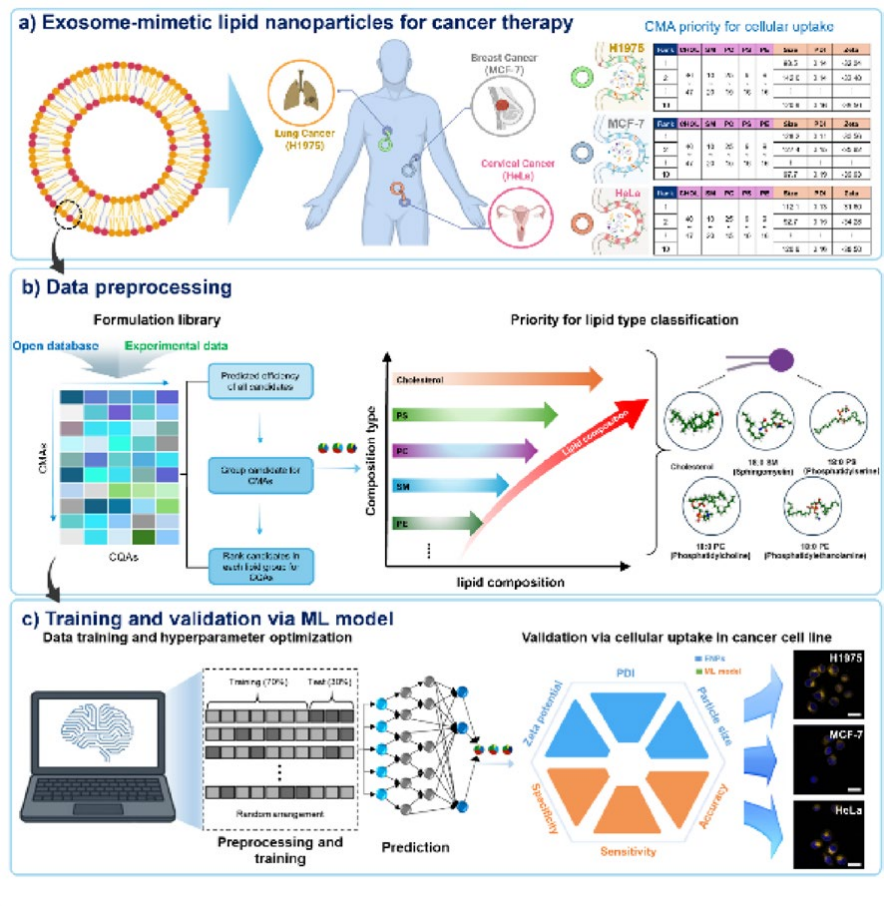
Schematic diagram of a hybrid algorithm that predicts the optimal composition of artificial exosomes. **a**. The optimal lipid nanoparticle compositions for lung, breast, and cervical cancer were identified and ranked (Top ten) using a hybrid algorithm, followed by the production of exosome formulations tailored to each cancer type. **b**. A normalized dataset was constructed by merging open databases and experimental formulation data. The top five candidate compounds for each lipid composition were selected as CMAs, and the evaluated CQA data were integrated to standardize the dataset structure. **c**. The datasets (17,800 composition parameters) were randomly split into training (70%) and blind test (30%) sets to train and validate the hybrid algorithm. The CQAs were then predicted and evaluated in terms of specificity, sensitivity, and accuracy. And then, cellular uptake assays were used to validate the optimal lipid composition for each cancer cell type. Created in BioRender. Park, S. (2025) https://BioRender.com/l4c0y7r

A hybrid algorithm was employed to capture complex, non-linear relationships between lipid compositions (Critical Material Attributes, CMAs) and ENP specification (Critical Quality Attributes, CQAs) for cellular uptake in three cancer cell lines in Fig. 1a. Data preprocessing and training dataset construction ~ 17,800 unique lipid composition–property pairs were compiled from open-access databases and experimental formulation data, as shown in Fig. 1b. Each formulation’s five most influential lipids define its CMAs. The corresponding CQAs, including size, polydispersity index (PDI), and zeta potential, were standardized across sources, and feature scaling normalized composition ratios to a range of 0 ~ 100. Outlier removal and class balancing via undersampling [31, 32] were performed to mitigate bias. The processed dataset was split into training (70%) and blind-test (30%) sets with 12,460 and 5,340 conditions, respectively.

The algorithm captures complex non-linear relationships between CMAs and CQAs, achieving high predictive performance (cross-validated R^2^ > 0.95) with high specificity and sensitivity (Fig. 1c). It predicts formulation performance and ranks the top 10 of ENP compositions for each cancer type. These top candidates were synthesized as artificial exosomes and validated in vitro (cellular uptake and cytotoxicity assays). The top formulations exhibited high cellular uptake (~ 95% in HeLa, ~ 92% in H1975, and ~ 91% in MCF-7), favorable physicochemical properties (diameter ~ 120 nm, polydispersity index < 0.2, and zeta potential ranging from − 40 to − 30 mV), and minimal toxicity, thereby corroborating the model’s predictions. Previous studies demonstrate that repeated administration of PEGylated nanomedicines induces anti-PEG immune responses, including the generation of anti-PEG IgM and complement activation. These responses trigger accelerated blood clearance (ABC), markedly decreasing circulation time and tumor uptake, thus limiting nanocarrier therapeutic efficacy [33]. Additionally, hypersensitivity reactions due to anti-PEG antibodies have been observed clinically [33]. These results support the investigation of our PEG-free, exosome-mimetic ENPs as a viable alternative. Although a direct comparison between ENPs and PEG-LNPs was not performed in this study, our approach is intended to bypass these immunological barriers and provide consistent therapeutic performance.

## Results

### Synthetic dataset expansion using lipidgan

The lipid formulation generative adversarial network (LipidGAN) generated 17,800 synthetic ENP compositions, representing roughly 80-fold expansion of the dataset (initial set of 225 experimentally-derived ENP compositions). The model effectively learned the underlying distribution of these five-component formulations and produced new samples that adhered to the same compositional patterns, thus expanding the collection without deviating from the realistic ENP design space. Model training converged after approximately 4,000 epochs, at which point we compiled the augmented set of formulations. This expanded dataset was randomly partitioned into a training set (70%) and a hold-out test set (30%) for downstream modeling (see Table 1 for details). By dramatically increasing the data volume, this GAN-driven augmentation provided much denser coverage of the composition space compared to the original sparse dataset. We anticipated that this enriched dataset would enable more robust training of the subsequent hybrid algorithm (Fig S1).

**Table 1:**
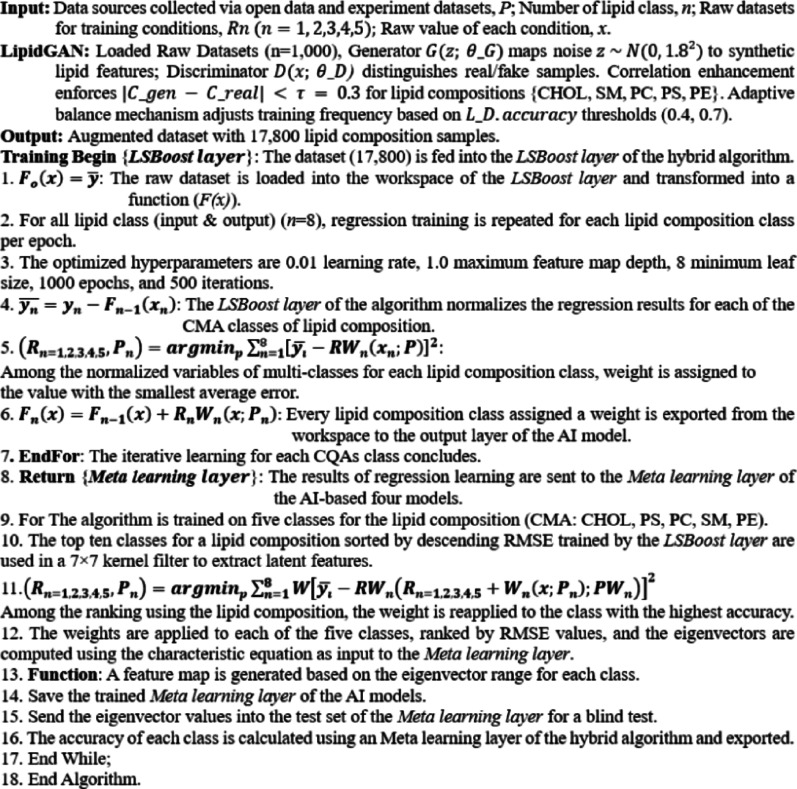
The training process of the hybrid algorithm & lipidgan model

To verify that the synthetic data represent real compositions, we compared the distribution of each lipid component in the GAN-generated formulations to that of the original experimental set. As shown in Supplementary Fig S2, the profiles of the synthetic formulations closely mirrored those of the original experimental set, with overlapping composition ranges and no significant deviations. The key findings are as follows: (1) Each of the five lipid components in the synthetic dataset closely matched its experimental data, which indicates the overall range of variability without bias. (2) The synthetic and experimental datasets demonstrated a high correlation (*R* = 0.88). (3) The mean absolute error was 0.10, and the root-mean-square error was 0.14 (see Supplementary Table S1). Especially, a correlation of 0.88 is considered strong by conventional standards [34], underscoring that the GAN-generated formulations successfully replicate the key patterns observed in the empirical data (Fig S3). No anomalies or artifacts were observed in the synthetic compositions, suggesting that LipidGAN captured the diverse latent features and variability present in the experimental dataset [35]. Collectively, these findings confirm that the augmented dataset is statistically similar to the original experimental dataset. Consistent with these observations, Table S2 shows that models trained solely on experimental data plateau at low predictive accuracy, likely due to the limited data available. In contrast, models augmented with LipidGAN-generated data achieve significantly higher and more robust predictive performance. Thereby providing a reliable foundation for the subsequent optimization of the hybrid algorithm’s artificial exosome composition. Thus, LipidGAN effectively addressed data scarcity by generating a large number of high-fidelity synthetic formulations for subsequent model development.

### Hybrid algorithm training and predictive performance

**Fig. 2:**
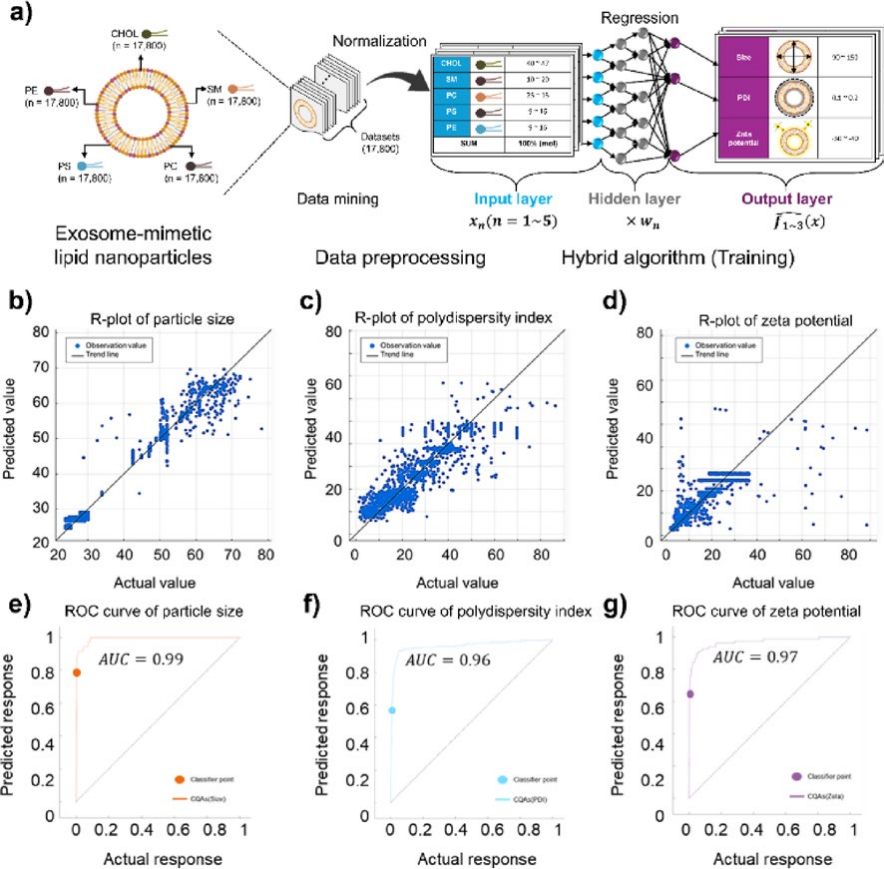
Workflow of the hybrid algorithm and its predictive performance for the optimal formulation of ENPs. **a**. Schematic workflow of the hybrid algorithm integrating regression learning with parallel feature extraction and automated weight allocation to identify optimal ENP compositions. A dataset comprising 17,800 formulations derived from five major lipid components (CHOL, SM, PC, PS, and PE) was pre-processed using compositional filtering, normalization, and categorization into CMAs. The algorithm was trained to predict three CQAs: particle size, PDI, and zeta potential. **b–d**. R-plots comparing the predicted and experimental values for size, PDI, and zeta potential. This yielded RMSE values of 0.01, 0.02, and 0.01, respectively, and R^2^ values of 0.99, 0.96, and 0.97, respectively. This confirms the algorithm’s high predictive accuracy for all CQAs. **e–g**. Receiver operating characteristic (ROC) curves and the corresponding area under the curve (AUC) values for the predictions of size (0.99), PDI (0.96), and zeta potential (0.97), demonstrating the algorithm’s excellent classification capability in distinguishing between high- and low-accuracy predictions for each CQA. Created in BioRender. Park, S. (2025) https://BioRender.com/l4c0y7r

We then trained a hybrid regression algorithm using the augmented dataset to predict key physicochemical attributes of the ENPs, including particle size, PDI, and zeta potential. Each candidate formulation was characterized by five major lipid components, with relative molar ratios summing to 100%. Using 12,460 formulations in the training set (70% of the augmented data), the algorithm learned how lipid composition influences these CQAs. The hybrid algorithm employs an ensemble-based parallel learning framework that dynamically adjusts feature weights and extracts underlying equations, enabling it to capture complex nonlinear relationships among the composition variables (Fig. 2a). Key hyperparameters included a leaf size of 8 and a learning rate of 0.02. The algorithm was trained for 1,000 epochs with 500 iterations per epoch (Table 1).

The trained algorithm exhibited an excellent fit to the data. It achieved a root-mean-square error (RMSE) of 0.01–0.02 and a coefficient of determination (R^2^) of 0.95–0.99 for the three CQAs of the ENPs (particle size, PDI, and zeta potential) on the training set (Fig. 2b–d). We further evaluated algorithm performance using receiver operating characteristic (ROC) curve analysis, where an area under the curve (AUC) approaching 1 indicates a highly accurate algorithm [36]. The algorithm’s ROC curves yielded AUC values of 0.99, 0.96, and 0.97 for size, PDI, and zeta potential, respectively (Fig. 2e–g), underscoring its robust predictive accuracy. These outstanding performances indicate minimal deviation between the algorithm’s predictions and the experimental values, confirming that the nonlinear mapping from lipid composition to each CQA was effectively learned (Tables S3 and S4).

### Blind test and correlation analysis of hybrid algorithms

**Fig. 3:**
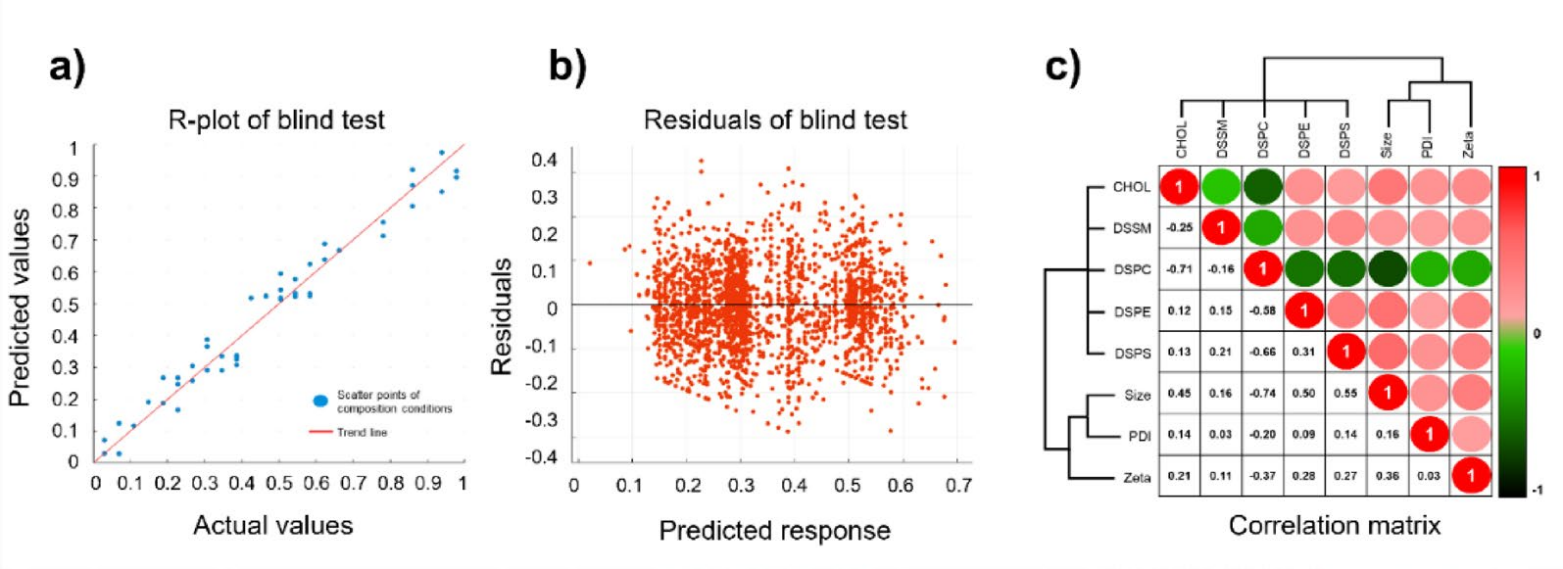
Blind test performance, residual error analysis, and correlation matrix for the hybrid algorithm’s optimal ENPs formulation. **a** A scatter plot comparing the predicted and experimentally measured CQAs for 5340 unseen ENP formulations (the 30% hold-out test set). The near-perfect correlation (RMSE = 0.02, R^2^ = 0.98) demonstrates the algorithm’s strong generalization capability and predictive accuracy under label-free conditions. **b** Residual distribution plot showing prediction errors centered symmetrically around zero, confirming the absence of systematic bias. Top ten-performing ENP formulations were identified based on their minimal Euclidean distances from the ideal 1:1 trend line. These were then further ranked using weighting coefficients from the algorithm’s loss function, prioritizing candidates with high prediction reliability. **c**. Correlation matrix of five CMAs and three CQAs, normalized using RMSE values. PC content exhibited the strongest negative correlations with multiple CQAs, indicating its dominant influence on ENP performance. This latent feature was extracted entirely under blind prediction conditions, thus validating the algorithm’s ability to capture the critical, biologically meaningful, nonlinear composition–function relationships for optimal formulation design

To evaluate generalization, we applied the trained algorithm to a test set comprising 5,340 previously unseen formulations (30% of the total data). In this label-free prediction scenario, the algorithm’s output predictions were directly compared to the corresponding experimental measurements (Fig. S5). The predicted values closely matched the observed outcomes, achieving a RMSE of 0.02 and a coefficient of determination (R^2^) of 0.98 (Fig. 3a). This level of accuracy on novel data underscores the algorithm’s strong predictive performance.

To identify the most promising ENP formulations, we singled out the ten compositions whose predicted outcomes lay closest (by Euclidean distance) to the ideal 1:1 trend line (Fig. 3a). In the blind test, the distribution of prediction residuals was symmetrically centered around zero, indicating unbiased performance without systematic error. These top candidates exhibited the smallest prediction errors, confirming the robustness of the hybrid algorithm. They were ranked by applying weighting coefficients from the algorithm’s loss function, thereby prioritizing formulations with minimal deviation and high prediction confidence. This residual-guided ranking approach illustrates the algorithm’s ability to capture complex nonlinear input–output relationships while minimizing variance in prediction error. It also enabled reliable identification of optimal formulations under label-free conditions (Fig. 3b).

We constructed a correlation matrix spanning the five CMAs and three CQAs to reveal latent interdependencies among the formulation components and quality attributes. In this matrix, values near + 1 (red) denote strong positive correlations, whereas values near − 1 (green) indicate strong negative correlations. Using an RMSE-normalized scale, we found that PC exhibited the most pronounced negative correlations with multiple CQAs, suggesting that this component exerts a dominant influence on ENP formation. This pattern emerged from the algorithm’s fully blind analysis, indicating that the algorithm autonomously learned key nonlinear relationships from the data. Moreover, the identification of PC as a crucial factor aligns with its known biological role in regulating membrane fluidity and cellular uptake, further validating that the algorithm captured meaningful composition–function dynamics essential for optimal ENP design (Fig. 3c). In a blind test, the hybrid algorithm proved robust and accurate under real-world, unlabeled conditions, successfully pinpointing the compositions most likely to exhibit optimal performance.

### Algorithm robustness and ablation study

**Fig. 4:**
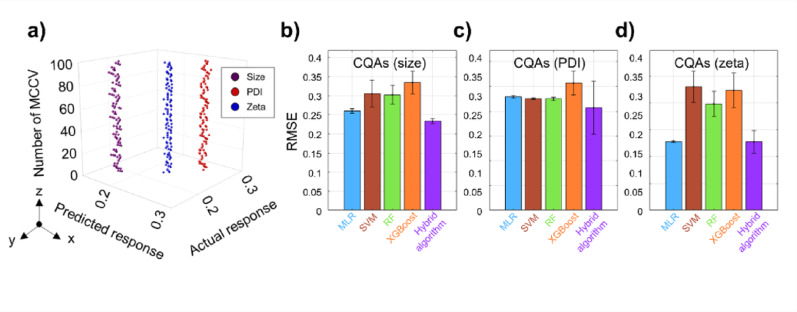
Validation and ablation study results of the hybrid algorithm. **a** Blind test results of the hybrid regression algorithm based on 100-fold MCCV. The plot illustrates the RMSE distribution for the three CQAs: particle size (purple), PDI (red), and zeta potential (blue), showing consistent trends across iterations. **b** Comparative RMSE analysis for particle size prediction across multiple algorithms, including MLR, SM, RF, and XGBoost, demonstrates that the hybrid algorithm achieved the lowest error, confirming its superior accuracy. **c** RMSE values from the ablation study for PDI prediction, highlighting performance differences between models. **d** RMSE comparison for zeta potential prediction among the same four models, further validating the hybrid algorithm’s enhanced predictive performance

Monte Carlo cross-validation (MCCV) was used to introduce further the hybrid algorithm’s reliability beyond the initial train–test evaluation. Specifically, 100 MCCV iterations were performed on the full dataset (*n* = 17,800) using stratified random splits (70% for training and 30% for testing). In each iteration, the model was retrained from scratch and used to predict all three CQAs for the held-out test set. The hybrid model consistently maintained high predictive accuracy across these trials, yielding a mean RMSE of ~ 0.07 and a mean R^2^ of ~ 0.92, with minimal variation between runs. Moreover, interrelationships among the predicted CQAs were preserved: pairwise R^2^ values between predicted size, PDI, and zeta potential remained above 0.98, and zeta potential predictions (blue points in Fig. 4a) showed only a marginally lower correlation with size and PDI (still R^2^ > 0.95). Importantly, these correlation patterns remained stable across repeated validations, indicating that the model captured complex composition–property relationships rather than overfitting. For example, increasing the proportion of specific lipid components consistently induced characteristic shifts in nanoparticle size, PDI, and zeta potential; these shifts in turn had cell–dependent effects on the identification of optimal ENP formulations. This mapping of composition to physicochemical outcomes underscores the algorithm’s capacity to guide rational formulation design without overfitting.

In a comparative evaluation (ablation study), the hybrid algorithm was benchmarked against several conventional regression methods: multiple linear regression (MLR), support vector machine (SVM), random forest (RF), and extreme gradient boosting (XGBoost). The hybrid model achieved the lowest RMSE for each CQA—0.23 for size (Fig. 4b), 0.04 for PDI (Fig. 4c), and 0.02 for zeta potential (Fig. 4d)—demonstrating an advantage in minimizing prediction errors across these diverse physicochemical properties. These results highlight the hybrid approach’s ability to capture nonlinear, multivariate relationships between lipid composition and each CQA, enabling it to outperform single-method models. Mean absolute error (MAE) and R^2^ metrics in Supplementary Table S5 similarly favor the hybrid model, providing additional evidence of its performance advantage.

### Physicochemical self-assembly mechanism of ENPs

The ENPs spontaneously self-assemble into stable vesicles ~ 120 nm in diameter, driven by fundamental physicochemical forces. The hydrophobic effect is the primary driver: amphiphilic lipids (with hydrophobic tails and hydrophilic heads) aggregate to minimize tail exposure to water [37]. They thus form a bilayer that closes into a spherical vesicle, eliminating any high-energy hydrophobic edges [38]. This closure is reinforced by the high line tension (γ) associated with exposed lipid-water interfaces, which strongly penalizes open edges and favors sealed spheres. Within the bilayer, van der Waals attractions between densely packed tails add cohesive energy, while hydrogen bonding among head groups (with water or cholesterol) further stabilizes the membrane. Electrostatic forces also modulate assembly: for instance, negatively charged lipids like PS cause head group repulsion that must be balanced by counterions or other lipids, influencing how tightly the bilayer packs [39]. Here, we introduce three theories, enabling the stable ENPs ~ 120 nm in diameter: Critical Packing Parameter (CPP), Gibbs free energy and Helfrich model.

The morphology of lipid self-assemblies is dictated by the molecular geometry of their amphiphilic constituents, particularly the relative dimensions of the hydrophilic headgroup and hydrophobic tail. This dependence is quantified by three geometric parameters—the hydrocarbon chain volume (*V*), the maximum effective chain length (*l*_*c*_), and the optimal headgroup surface area (*a*_*0*_, the area at the lipid–water interface)—which together define the dimensionless critical packing parameter (CPP) as shown in Eq. (1):

1 $$\:CPP=\frac{V}{{a}_{0}{l}_{c}}$$

The CPP predicts the structure that lipids will self-assemble into; lipids with CPP < ~ 1/3 adopt a cone-like shape, favoring spherical micelles; those with 1/3 < CPP < 1 form truncated cone or cylindrical shapes (yielding curved bilayers or cylindrical micelles); CPP ≈ 1 produces planar bilayers; and CPP > 1 corresponds to inverted-cone shapes forming inverse micelles. Consistent with these expectations, our experimental results showed that formulations with CPP < 0.5 produced micelles, those with 0.5–1 formed flexible bilayer vesicles, CPP ~ 1 yielded stable planar bilayers, and CPP > 1 gave inverse micellar structures. These findings confirm that CPP is a robust predictor of lipid self-assembly behavior and provide a practical design guideline for engineering exosome-mimetic nanoparticles (ENPs) with specific morphologies [40]. However, CPP alone did not account for the size of the assemblies: even within the 0.5–1 CPP range, the diameters of resulting ENPs varied from ~ 50 to 150 nm. This variability in size was influenced by additional factors, such as membrane bending energy and thermodynamic stability. In particular, minimizing Gibbs free energy played a key role in determining both vesicle size distribution and morphological stability [41].

The self-assembly and eventual size of extruded lipid nanoparticles (ENPs) are fundamentally governed by the minimization of Gibbs free energy. The Gibbs free energy can be calculated using Eq. (2):

2 $$ \Delta {\text{G = }}\Delta {\mathrm{H}} - T\Delta S $$

Enthalpic contributions (*ΔH*) include the favorable energetic gain from hydrophobic lipid–lipid interactions (e.g., van der Waals packing of acyl chains and removal of lipid–water contacts) as well as the cost of bending a flat bilayer into a curved vesicle. By contrast, the entropic term (*TΔS*) is dominated by the hydrophobic effect – water molecules that were previously ordered around the lipids’ hydrophobic tails are released upon bilayer formation, greatly increasing the system’s entropy [41]. Importantly, vesicle curvature (which varies with radius) alters this enthalpy–entropy balance. A smaller vesicle (< 100 nm radius) has a higher curvature and thus incurs a larger bending-energy penalty (raising *ΔH*), whereas a larger vesicle (≈ 120 nm radius) has a lower curvature cost (lower *ΔH*) but yields little additional entropy gain once the bilayer is closed [42]. In this regard, *ΔG* reaches a minimum at an intermediate vesicle size where these opposing factors balance, corresponding to a thermodynamically stable ENP radius [43]. In other words, any deviation from this optimal size would increase *ΔH* or reduce *TΔS*, driving the vesicle back toward the free-energy minimum. This thermodynamic balance thereby stabilizes ENPs around their equilibrium size and sets the stage for a more quantitative description of the curvature energy via the Helfrich model.

The vesicle’s curvature is governed by membrane bending mechanics [44]. In the Helfrich model, the energetic cost of deforming a bilayer depends on its bending rigidity (*k*) and spontaneous curvature (*c*_0_), the intrinsic curvature at which the membrane is most stable. The bending energy can be expressed as the Eq. (3):

3 $$ E_{{bend}} = \frac{1}{2}\kappa \cdot \left( {c_{1} + c_{2} - c_{0} } \right)^{2} + \bar{\kappa } \cdot c_{1} \cdot c_{2} $$

where c_1_ and c_2_ are the principal curvatures of the surface, and $\bar{k}$ is the Gaussian curvature modulus. A membrane with *c*_0_ ≈ 0 and large k (stiff) resists bending and thus favors a flatter shape (large radius of curvature). Conversely, if the lipid composition imposes a non-zero *c*_0_ or lowers *k*, the bilayer can curve into a smaller vesicle with less energy penalty.

Lipid composition plays a key role in curvature accommodation and final particle size. Membranes rich in SM and cholesterol (as in natural exosomes) tend to have *c*_0_ ≈ 0 and high k, rendering them rigid and less able to sharply curve. The vesicles from such stiff compositions thus favor large diameters (lower curvature) to minimize bending stress. Notably, the *γ* at domain boundaries or defects provides a different energetic incentive: it penalizes the presence of any boundary line between two coexisting phases. A high *γ* drives the system to eliminate domain interfaces by merging domains or physically budding one domain away from the other to reduce the shared boundary length [45]. Incorporating lipids with cone-shaped geometry or negative charge (e.g., phosphatidylethanolamine (PE, small head group) or anionic PS) biases the membrane toward higher curvature. These components either intrinsically prefer negative curvature (PE curves the bilayer inward) or introduce head group repulsion relieved by greater curvature (more area per lipid). Consequently, PE/PS-enriched ENPs stabilize at smaller vesicle sizes, as these curvature-friendly lipids lower the bending energy cost at a given small radius.

Ultimately, the ENPs settle at a size that balances these forces and bending energies. After extrusion through 150 nm pores, vesicles relax to an optimal ~ 120 nm diameter where the total membrane free energy is minimized. At this scale, the bending energy is low enough to maintain the structure, and energy penalties oppose any tendency to grow or shrink. Smaller vesicles (higher curvature) would cause steep bending costs (especially for rigid compositions), while much larger vesicles could become unstable or fragment under agitation [46]. Thus, ~ 120 nm represents a local free-energy minimum: this size eliminates line-tension costs (no edges) and balances bending rigidity with the lipids’ intrinsic curvature tendencies. This mechanism explains why the ENPs remain monodisperse near 100–120 nm, yielding stable nanoscale carriers for drug delivery.

### Cellular uptake efficiency and biocompatibility of ML-driven ENP formulations

**Fig. 5:**
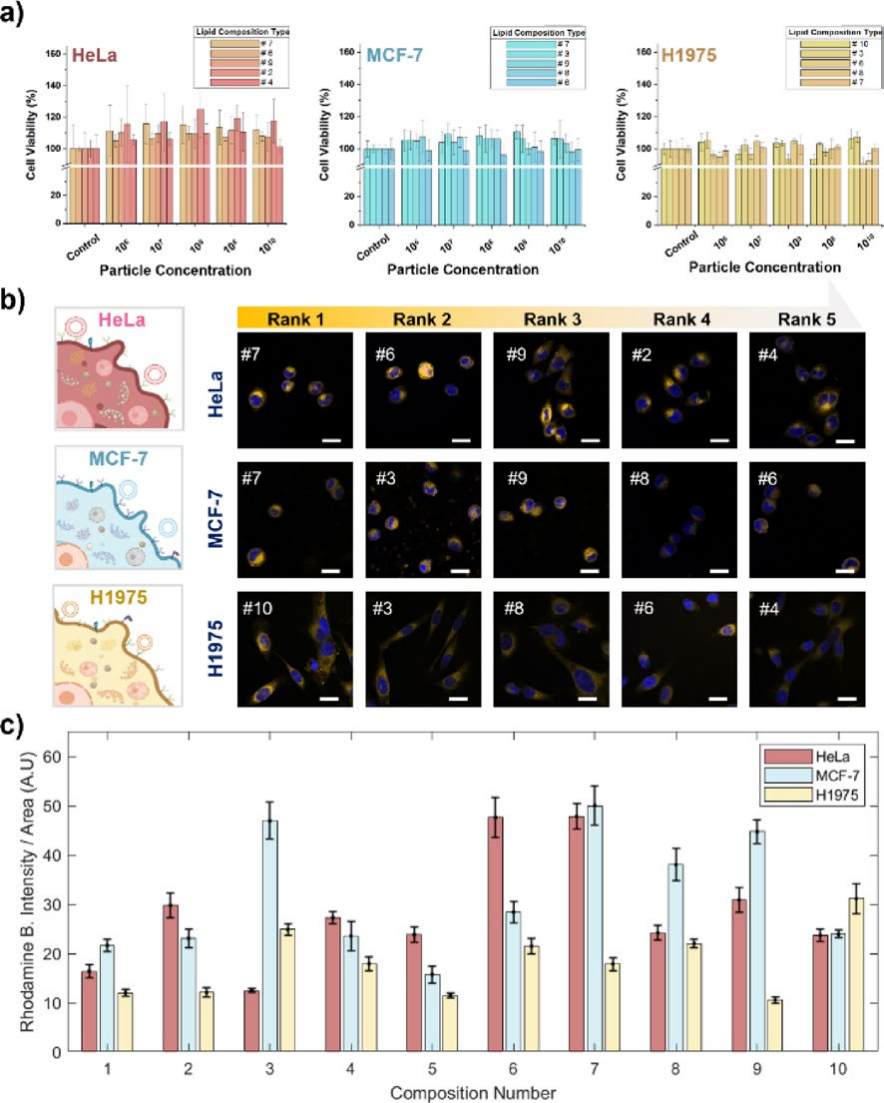
Cellular uptake efficiency and biocompatibility of ML-driven ENP formulations. **a**. Cell viability of H1975, MCF-7, and HeLa cells after 24 h exposure to ten different ENP formulations at doses up to 1 × 10^10^ particles per well, measured using a CCK-8 assay. All formulations maintained > 90% viability across all cell lines, with the top five formulations for each cell type ranked and shown. Data represent mean ± SD (*n* = 4). **b**. Confocal fluorescence microscopy images of the top five ENP formulations in each cell line (1 × 10^10^ particles/well) after 8 h incubation. ENPs were labeled with rhodamine B (red, λ_ex_ = 545 nm, λ_em_ = 545 nm), and nuclei were stained with DAPI (blue). Scale bars: 20 μm. Intense perinuclear red fluorescence indicates successful intracellular internalization of ENPs. **c**. Bar graph showing the quantitative analysis of intracellular rhodamine B fluorescence intensity per cell, obtained from confocal images using ImageJ. Data represent mean ± SEM (*n* = 3). Higher fluorescence intensity indicates greater ENP uptake efficiency. Formulation 6 was consistently ranked within the top five across all three cell types, demonstrating high internalization efficiency. Created in BioRender. Park, S. (2025) https://BioRender.com/l4c0y7r

We first evaluated the cytotoxicity of the ten ML–driven ENP formulations in three human cell lines (HeLa, MCF-7, and H1975) to confirm biocompatibility. Cells were exposed to each formulation at five dose levels (up to 1 × 10^10^ particles per well), and viability was assessed after 24 h using a CCK-8 assay. All formulations exhibited high cell viability (≥ 90%, typically 95–100%) at all doses in all cell lines, with no significant differences from untreated controls (*p* > 0.05; Supplementary Tables S6–S9). At the highest particle dose, the five formulations yielding the greatest cell viability were 10, 3, 6, 8, and 7 in H1975 cells; 7, 3, 9, 8, and 6 in MCF-7 cells; and 7, 6, 9, 2, and 4 in HeLa cells. These top-performing formulations are highlighted in Fig. 5a, and complete viability data for all formulations are presented in Supplementary Fig S6. Based on these results, we designated 1 × 10^10^ particles per well as a non-cytotoxic dose for subsequent studies.

Next, we examined cellular uptake at the non-cytotoxic dose of 1 × 10^10^ particles per well. Confocal micrographs (Fig. 5b) taken after 8 h of incubation with rhodamine B-labeled ENPs revealed robust nanoparticle internalization in all three cell lines. Red fluorescence from the labeled particles was observed throughout the cytoplasm, with a predominantly perinuclear distribution around the DAPI-stained nuclei (blue), confirming successful ENP uptake. For clarity, Fig. 5b shows only the five formulations with the highest uptake in each cell line, while images for all formulations are presented in Supplementary Figs S7–S9. These top-performing formulations exhibited intense intracellular fluorescence, underscoring efficient nanoparticle uptake.

To quantify ENP uptake, confocal micrographs were analyzed with ImageJ to measure intracellular rhodamine B fluorescence per cell (Fig. 5c, Fig. S10). Uptake efficiency varied markedly among formulations and across cell types. In H1975 cells, formulations 10, 3, 8, 6, and 4 yielded the highest uptake. MCF-7 cells showed the most significant uptake with formulations 7, 3, 9, 8, and 6. Similarly, HeLa cells exhibited the strongest signals with formulations 7, 6, 9, 2, and 4. Notably, formulation 6 was consistently among the top five in all three cell lines, indicating high internalization efficiency across cell types.

Compositional analysis of the high-uptake ENP formulations revealed common features likely underlying their performance. Most top-ranked formulations contained elevated cholesterol (≥ 46%) along with intermediate levels of phosphatidylcholine (PC, ~ 14–20%) and phosphatidylethanolamine (PE, ~ 12–16%). This trend was evident in MCF-7 and HeLa cells: formulations with PC content ≥ 19% showed enhanced uptake, consistent with Fig. 3c, where PC is identified as a key determinant of ENP uptake. In contrast, H1975 cells exhibited strong uptake even with lower cholesterol (40–41%) in some formulations, suggesting that PS and relative PE content also influence uptake in a cell-type-dependent manner. These lipid compositions likely promote stable, fluid nanoparticle membranes that favor membrane fusion or endocytosis, thereby enhancing cellular uptake [47–49]. Collectively, these compositional features likely explain the superior uptake of these formulations.

Finally, ENP uptake was cell-type dependent: MCF-7 cells internalized the nanoparticles most efficiently, followed by HeLa cells, with H1975 cells showing the lowest uptake (MCF-7 > HeLa > H1975). This trend suggests inherent differences in endocytic capacity and membrane properties among the cell lines. Nonetheless, all ML-driven ENP formulations were highly biocompatible and achieved robust intracellular delivery in all three cell lines.

### Discussion

A potential concern with removing cationic lipids is that the resulting ENPs carry a net negative charge, which may affect cellular interactions. However, evidence suggests that negatively charged nanoparticles can still internalize efficiently into cancer cells. In vitro studies demonstrate that the surface charge (positive or negative) has a significant influence on uptake pathways and rates [50]. In some cases, anionic liposomes and nanoparticles achieve uptake comparable to or even higher than their cationic counterparts [51, 52]. Harush-Frenkel et al. observed distinct endocytic routes for oppositely charged nanoparticles in HeLa cells: anionic particles entered via non-clathrin pathways [53]. Similarly, He et al. found that both positively and negatively charged polymeric nanoparticles were taken up by macrophages more readily than neutral particles [54]. Notably, anionic liposome systems also demonstrate greater serum stability and lower toxicity than cationic systems [51]. These studies challenge the assumption that a cationic surface is required for effective delivery. They indicate that anionic ENPs can achieve endogenous uptake mechanisms while avoiding the pitfalls of cationic carriers. Likewise, Ochoa-Sánchez et al. recently highlighted anionic liposomes as promising alternatives, citing reduced immune recognition and prolonged circulation relative to conventional cationic liposomes [51].

In this study, we developed a hybrid algorithm for predicting artificial exosome composition. We systematically validated it via ablation analysis against conventional regression models, including MLR, SVM, RF, and XGBoost, evaluating CQAs. The hybrid approach consistently outperformed all baseline models, yielding the lowest RMSE and MAE values, as well as the highest R^2^ for each CQA. These results support the development of PEG-free, negatively charged ENPs as a new class of biocompatible nanocarriers for chemotherapy. Additionally, ENPs are protein-free vesicles formulated to match the lipid composition of exosomes. This lipid-only design enables ENPs to use optimal lipid properties for cellular uptake via membrane fusion and endocytosis. Whereas native exosomes use surface proteins for receptor-ligand targeting, ENPs instead rely on these lipid-based mechanisms. As a result, the lipid-only approach yields high batch-to-batch reproducibility, which is critical for therapeutic applications. Further analysis revealed a strong association between cellular uptake specificity and the PC content of each ENP formulation. The hybrid algorithm identified PC-enriched compositions optimized for HeLa, H1975, and MCF-7 cells. These PC-rich formulations achieved high uptake (91–95%) and maintained viability of greater than 90% across all three cell lines. Notably, PC content exhibited the strongest correlation with all CQAs, highlighting its central role in the predictive algorithm (Fig. 3c).

Zhan et al. demonstrated a similar effect: tumor cell internalization doubled when exosomal membranes were enriched with PC [54]. They attributed this enhancement to strong interactions between PC components in the exosome membrane and the tumor cell membrane. Their experiments further showed that uptake efficiency increased with PC concentration up to 10–40 µg, beyond which membrane integrity was compromised and internalization declined [54]. These observations align with the label-free predictions of our hybrid algorithm: the predicted ENP compositions closely resemble natural exosome lipid profiles, particularly in their high PC content. Thus, PC likely modulates membrane fluidity and curvature, facilitating selective uptake and improving therapeutic compatibility in cancer cells.

Interestingly, although PC enrichment was the dominant determinant of uptake across all cell types tested, uptake patterns depended on cell type specificities. While HeLa and MCF-7 cells internalized cholesterol- and PC-rich formulations, consistent with membrane fusion-mediated uptake, H1975 cells (a non-small cell lung cancer line) also showed uptake patterns associated with PS (See Supplementary Fig S11). Previous studies have shown that NSCLC cells, including H1975, upregulate the PS-binding receptor TIM-4 in response to inflammatory cues such as IL-6, TNF-α, TGF-β, and LPS [55]. Moreover, PS-recognizing receptors, such as TIM-4 and Stabilin-2, are well-characterized mediators of the efficient internalization of PS-exposing vesicles [56, 57]. Our findings suggest that while PC is the central driver of ENP uptake, additional lipid–receptor interactions—particularly PS–TIM-4 and PS-Stabilin-2 binding—can further enhance uptake in a cell-type-specific manner.

Distinct functions of lipid components indicate distinct uptake patterns. Cholesterol adjusts bilayer fluidity and lowers the energy required for fusion pore formation, enhancing cellular uptake [58]. Conical-shaped PE causes negative membrane curvature, promoting membrane insertion and endocytosis [59]. PS is recognized by specific receptors and promotes macropinocytosis, which correlates with increased uptake of PS-enriched formulations in H1975 cells [55–57]. Together, these lipid-specific mechanisms explain the trends identified by our machine learning model. Still, the model only uses physicochemical and in vitro uptake data, without accounting for in vivo complexities such as protein corona formation or immune clearance. Also, lipid components were limited to five major classes for feasibility. To address the translational potential, further studies on AI-powered active pharmaceutical ingredients (APIs) is required to evaluate drug encapsulation efficiency and therapeutic efficacy in cancer models.

## Conclusions

This work integrates biomimetic nanotechnology with an ML-driven optimization strategy to develop a next-generation chemotherapy delivery platform. The hybrid algorithm achieved high predictive accuracy (with a coefficient of determination R^2^ > 0.95 on validation sets), indicating an excellent fit between predicted and observed nanoparticle characteristics. Using this algorithm, we performed an in silico screen to identify the top-ranked ENP formulations optimized for cellular uptake efficiency and favorable physicochemical properties. We proceeded to synthesize and test the top ML-recommended ENPs in vitro using three representative human cancer cell lines: HeLa (cervical carcinoma), H1975 (lung carcinoma), and MCF-7 (breast adenocarcinoma). The results exhibited robust uptake (~ 91–95% after 8 h) across all tested cancer cells. PC enrichment emerged as the principal lipid determinant of nanoparticle uptake, as indicated by correlation analysis. Formulations with high PC content—combined with cholesterol (≥ 46%), balanced levels of phosphatidylethanolamine, and contributions from phosphatidylserine—consistently promoted efficient nanoparticle internalization.

These ENPs exhibited an average hydrodynamic diameter of approximately 120 nm (suitable for tumor penetration), a low PDI (< 0.2, indicating a uniform size distribution), and a mildly negative zeta potential (− 30 to − 40 mV). This degree of negative surface charge is sufficient to maintain colloidal stability without inducing aggregation (Supplementary Table S9, Figs S12-S13). Moreover, the ENPs showed minimal cytotoxicity toward healthy cells and caused no significant acute toxicity in our studies, consistent with their inherent biocompatibility. In this regard, these findings demonstrate that our machine learning–driven ENP formulation can overcome the long-standing trade-off between efficacy and safety in nanoparticle drug delivery. Consequently, this study introduces a promising nanomedicine platform for translational cancer therapy and exemplifies a paradigm shift for accelerating nanotherapeutic development by integrating machine learning with rational nanomaterial design.

## Methods/experimental

### Materials

1,2-distearoyl-sn-glycero-3-phosphocholine (DSPC), 1,2-distearoyl-sn-glycero-3-phospho-L-serine (DSPS), N-stearoyl-D-erythro-sphingosylphosphorylcholine (DSSM), 1,2-distearoyl-sn-glycero-3-phosphoethanolamine (DSPE), and was purchased from Avanti Polar Lipids (USA). Cholesterol was purchased from Sigma-Aldrich (USA).

### Preparation of exosomal nanoparticles

All nanoparticles were prepared using the thin-film hydration method. DSPC, DSPS, DSSM, DSPE, and cholesterol, corresponding to each composition, were dissolved in a chloroform/methanol mixture (2:1, v/v). The organic solvents were evaporated under a stream of nitrogen gas and subsequently removed by vacuum drying for 30 min. The resulting dried lipid films (1 mg total lipid) were hydrated in 1 mL of distilled water (yielding a lipid-to-aqueous ratio of 1 mg/mL) and vortexed vigorously for 5 min. The hydrated lipid solution was sonicated three times for 30 min each, with 10 min intervals, using a bath-type sonicator (POWERSONIC 505, Hwashin Tech). During sonication, the temperature of the water bath was maintained below 30 °C. After sonication, the lipid nanoparticles were extruded 10 times sequentially through polycarbonate membranes with pore sizes of 200 nm and 100 nm using a mini-extruder (Avanti Polar Lipids, Inc.) to produce exosomal nanoparticles with the desired compositions (the detailed manufacturing process is illustrated in Fig S4, and lipid formulations are listed in Supplementary Tables S3 and S4).

### Cell culture

HeLa, MCF-7, and H1975 cell lines were obtained from the American Type Culture Collection (ATCC, USA). HeLa and MCF-7 cells were cultured in Dulbecco’s Modified Eagle Medium (DMEM; Gibco, USA), while H1975 cells were maintained in Roswell Park Memorial Institute (RPMI) 1640 medium (Gibco, USA). All media were supplemented with 10% fetal bovine serum (FBS) and 100 U/mL penicillin–streptomycin. Cells were incubated at 37 °C in a humidified atmosphere containing 5% CO_2_ until they reached the appropriate confluency for subculturing.

### Characterization of nanoparticles

The particle size distribution and number density of the exosomal nanoparticles were analyzed using nanoparticle tracking analysis (NTA) with an LM10 instrument (Malvern Instruments, Malvern, UK), and the data were processed using the corresponding NTA software. Samples were diluted in distilled water according to NTA guidelines to achieve optimal particle concentration. In addition, the average particle size, PDI, and zeta potential were measured using a particle size and zeta potential analyzer (Otsuka Electronics, Osaka, Japan), following the manufacturer’s instructions. For morphological analysis, 10 µL of the nanoparticle suspension was placed onto formvar/carbon-coated copper grids and incubated for 15 min to allow adsorption. The grids were then negatively stained with UranyLess EM solution (Electron Microscopy Sciences, Hatfield, PA, USA) for 15 min and air-dried overnight. The stained samples were examined using a transmission electron microscope (JEM-2100Plus; JEOL, Tokyo, Japan).

### In vitro cytotoxicity assay

Cytotoxicity was evaluated using the Cell Counting Kit-8 (CCK-8; GlpBio, USA) assay in MCF-7, HeLa, and H1975 cells treated with exosomal nanoparticles of different lipid compositions. Each cell line was seeded at a density of 1 × 10⁴ cells per well in a 96-well plate and incubated at 37 °C for 24 h. After incubation, the culture medium was removed, and the wells were washed twice with Phosphate Buffered Saline (PBS). The cells were then treated with various concentrations of each composition of exosomal nanoparticles in fresh culture medium and incubated for an additional 24 h. Following treatment, the medium was discarded, the wells were washed with PBS, and 100 µL of culture medium containing 10% CCK-8 reagent was added to each well. The cells were incubated for 1 h at 37 °C, and absorbance was measured at 450 nm using a microplate reader (TECAN Infinite^®^ 200 PRO; TECAN Group Ltd., Switzerland). Relative cell viability was calculated using the following Eq. (4).

4 $$\:Cell\:viability\:\left(\%\right)=\:\frac{({Ab}_{s}-{Ab}_{b})}{({Ab}_{c}-{Ab}_{b})}\times\:100$$

where $\:{Ab}_{s}$, $\:{Ab}_{c}$, $\:{Ab}_{b}$ represent the absorbance of the sample, control, and blank wells, respectively.

### In vitro cellular uptake assay

For confocal microscopy analysis, MCF-7, HeLa, and H1975 cells were seeded in 6-well culture slides at a density of 1 × 10^5^ cells per well and incubated at 37 °C. The cells were then washed with PBS, and fresh medium containing 1 × 10^10^ ENPs of each lipid composition, labeled with DOPE-Rho B (0.1 mol%), was added to each well. After 8 h of incubation, the cells were washed again with PBS and fixed with 2% paraformaldehyde (PFA) solution. The nuclei were counterstained with DAPI mounting medium (Vector Laboratories, CA, USA). Internalization of the nanoparticles was visualized using a confocal laser scanning microscope (LSM 900; Carl Zeiss, Germany), and fluorescence intensity per cell was quantified using ImageJ software (NIH, USA).

### Dataset Preparation and preprocessing

The top five lipid components were used to generate a total of 17,800 artificial exosome formulation rows. Each entry consisted of normalized molar ratios summing to 100. The dataset was randomly split into 70% training datasets (12,460) and 30% blind test datasets (5,340). Prior to training the algorithm, all data were pre-processed using normalization and scaling to ensure compatibility with the hybrid algorithm regression.

### Hybrid algorithm architecture

The proposed hybrid algorithm is structured around an ensemble-based regression approach that integrates parallel learning layers. Least squares boosting (LSBoost) layer as the core regression model, supported by an ensemble framework capable of handling non-linear relationships between lipid compositions and CQAs such as particle size, PDI, and zeta potential. This architecture is further enhanced by automatic weight allocation mechanisms and dynamic feature equation extraction functions to strengthen variable interaction analysis.

### Regression learning with feature equation extraction

The regression process employs a sequential ensemble learning strategy where predictive functions are updated at each stage as follows. The prediction function is calculated using Eq. (5).

5 $$ F_{n} \left( x \right)~ = ~F_{{n - 1}} \left( x \right)~ + ~y_{n} ~\cdot~W_{n} \left( {x,~E_{n} } \right) $$

Here, $y_{n}$is the weighted update value derived from normalized RMSE-based ranking. The final ranking is computed and assigned by Eq. (6):

6 $$ y_{n} = \frac{{\left( {\sqrt {\left( {\left( {\frac{1}{n}} \right)} \right.~ \cdot \sum\nolimits_{i} { = 1^{n} } } \left( {\widehat{{y_{i} }}~ - ~y_{i} } \right)^{2} } \right) - ~\left. {RMSE_{{\min }} } \right)}}{{\left( {RMSE_{{\max }} - RMSE_{{\min }} } \right)}} $$

The LSBoost algorithm assigns higher weights to samples that are difficult to predict, thus improving model accuracy iteratively.

### Model evaluation metrics

To evaluate the predictive performance of the regression model, standard statistical metrics were used. The MAE was calculated using Eq. (7), the MSE using Eq. (8), and the RMSE using Eq. (9).

7 $$\:MAE=\:\frac{1}{n}{\sum\:}_{i=1}^{n}\left|\widehat{{y}_{i}}-{y}_{i}\right|$$

8 $$\:MSE=\:\frac{1}{n}{\sum\:}_{i=1}^{n}{\left(\widehat{{y}_{i}}-{y}_{i}\right)}^{2}$$

9 $$\:RMSE=\:\sqrt{\frac{1}{n}{\sum\:}_{i=1}^{n}{\left(\widehat{{y}_{i}}-{y}_{i}\right)}^{2}}$$

### Correlation analysis and residual distribution

The correlation between predicted and actual values was quantified using the coefficient of determination. The coefficient (R^2^) was calculated using Eq. (10).

10 $$ R^{2} = ~\frac{{\sum \left( {x_{i} - \bar{x}} \right)\left( {y_{i} - \bar{y}} \right)}}{{\sqrt {\sum \left( {x_{i} - \bar{x}} \right)^{2} \sum \left( {y_{i} - \bar{y}} \right)^{2} } }} $$

Residuals $e_{i} = y_{i} - \hat{y}_{i}$ were analyzed to assess deviation patterns and verify that prediction errors followed a normal distribution without systemic bias.

### Monte carlo cross-validation (MCCV)

To evaluate the robustness of the hybrid regression model, MCCV was conducted over 100 iterations using stratified random splits (70% training, 30% blind test). The overall prediction error across the results of the blind test was computed by using Eq. (11):

11 $$\:{\epsilon\:}_{MCCV}\:\left(\%\right)=\frac{1}{100}{\sum\:}_{i=1}^{100}{\epsilon\:}_{i}$$

All computations were performed in MATLAB R2023b on a workstation with an Intel Core i9-12900KS CPU (3.40 GHz), 32GB RAM, and an NVIDIA GeForce RTX 3090 Ti GPU.

## Supplementary Information

Below is the link to the electronic supplementary material.

Supplementary Material 1

## Data Availability

The dataset used in this study, comprising lipid composition condition lists for pre-training and blind testing of the hybrid algorithm, is available at Zenodo (10.5281/zenodo.16928013), and the machine-learning codes developed and tested in the MATLAB 2023b environment are available at GitHub (https://github.com/SeongminHA/Machine-learning-driven-rational-design-of-exosome-mimetic-lipid-nanoparticles-for-cancer-therapy), with access provided upon reasonable request.
