# Supplementary material for: Machine learning-driven exosome-mimetic lipid nanoparticles for tumor-specific targeting
Source: Nano Converg. 2026 Jan 28;13:2. doi: 10.1186/s40580-026-00531-7 (PMC12852563; doi:10.1186/s40580-026-00531-7)
Supplement: Supplementary file 1 — Supplementary Material 1 [file 40580_2026_531_MOESM1_ESM.docx]

**Supplementary Material**

**Machine learning-driven exosome-mimetic lipid nanoparticles for tumor-specific targeting**

Seongmin Ha ^1 †^, Do Hyun Lee ^2 †^, Taehoon Lee ^1 †^, Hairi Jiang ^1 †^, Hyun-jin Lee ^2^, Seungbum Seo ^1^, Ji Yeong Yang ^1, 2^, Sunyoung Park ^3^, Sung-Gyu Park ^4^, Joonchul Shin ^4 *^, and Hyo-Il Jung ^1, 2, 5 *^

^1^ School of Mechanical Engineering, Yonsei University, 50 Yonsei-ro, Seodaemun-gu, Seoul, 120-749, Republic of Korea.

^2^ The DABOM Inc., 50 Yonsei-ro, Seodaemun-gu, Seoul, 120-749, Republic of Korea.

^3^ Department of Biomedical Technology, Kangwon National University, Republic of Korea.

^4^ Advanced Bio and Healthcare Materials Research Division, Korea Institute of Materials Science (KIMS), Changwon, Gyeongnam, Republic of Korea.

^5^ Department of Integrated Medicine, Yonsei University, 50 Yonsei-ro, Seodaemun-gu, Seoul, 120-749, Republic of Korea.

^†^ These authors contributed equally to this work

* Corresponding author: Joonchul Shin, Hyo-Il Jung

E-mail address: Joonchul Shin (jcs2078@kims.re.kr), and Hyo-Il Jung (uridle7@yonsei.ac.kr)


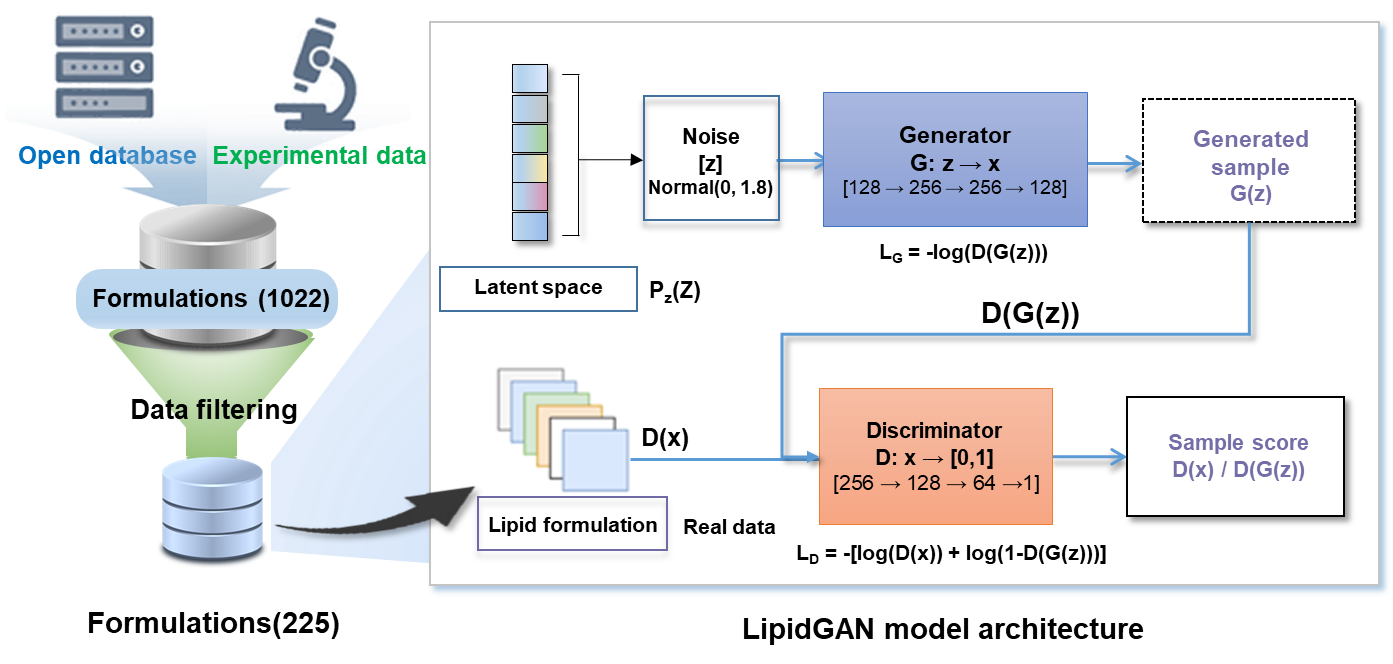


**Fig S1. Architecture of the LipidGAN model.** The workflow shows data collection from open databases and experimental data (1,022 formulations), followed by filtering to obtain 225 high-quality lipid formulations for training. The GAN architecture consists of a Generator (G) that transforms random noise z from a 64-dimensional latent space into synthetic formulation data, and a Discriminator (D) that distinguishes between real and generated samples. The Generator processes noise through progressive neural network layers (128 → 256 → 256 → 128 neurons) to produce 8-dimensional output vectors containing five lipid composition features (CHOL, SM, PC, PS, PE) and three physicochemical properties (particle size, PDI, zeta potential). The Discriminator uses a contracting architecture (256 → 128 → 64 → 1 neurons) to output probability scores. The adversarial training employs competing loss functions: LG = -log(D(G(z))) for the generator and LD = -[log(D(x)) + log(1-D(G(z)))] for the discriminator, enabling generation of novel lipid formulations while preserving original data distributions and inter-feature correlations.

**
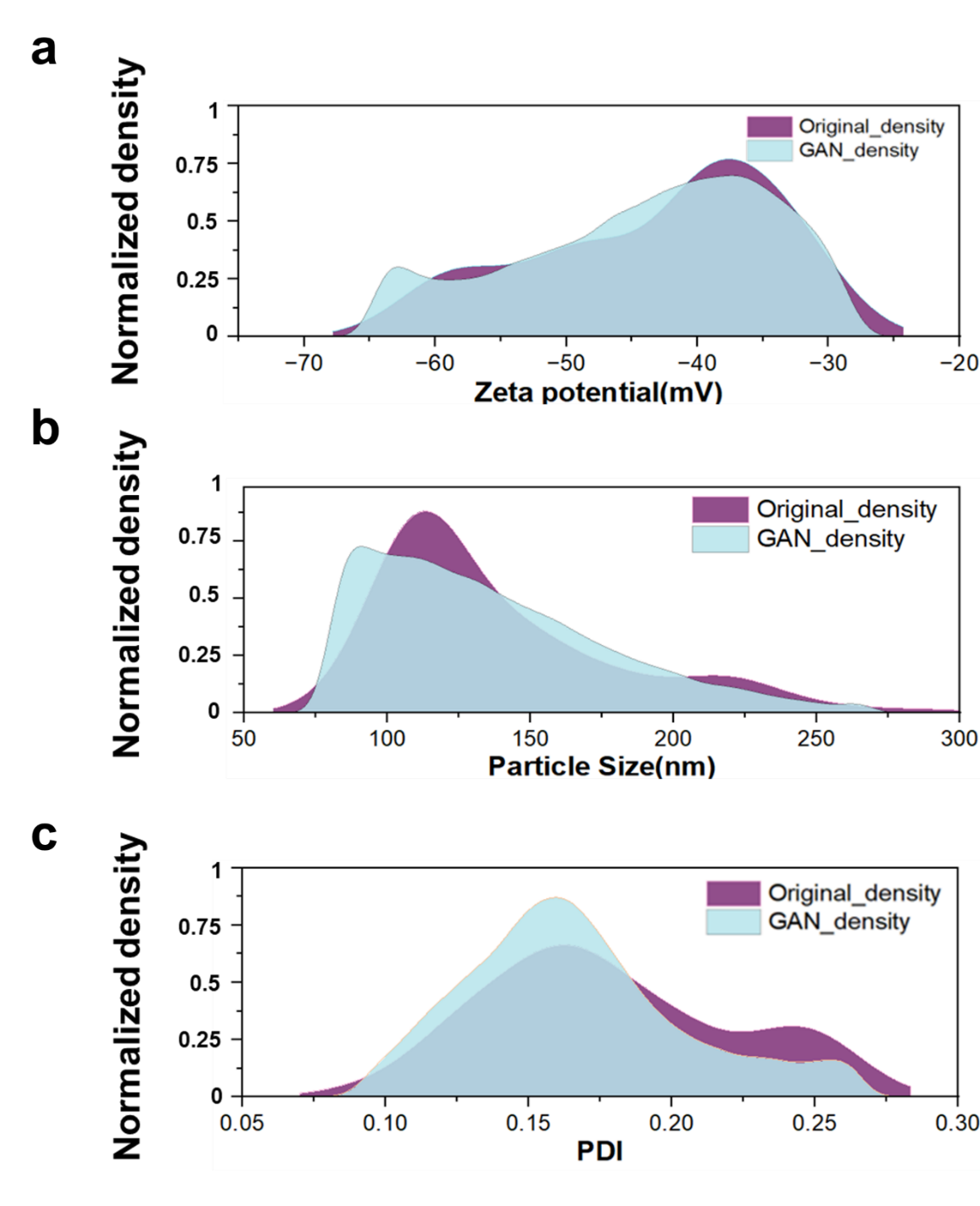
Fig S2. Comparative analysis of normalized density distributions.** The figure presents a visual comparison between the probability density distributions of the original experimental data (purple area) and the synthetic data generated by the Generative Adversarial Network (GAN) model (light blue area). The comparison is shown for three critical physicochemical properties: (a) Particle Size: 81% similarity, (b) Polydispersity Index: 89% similarity, and (c) Zeta potential: 87% similarity. To enhance visual clarity and facilitate the comparison of distributional shapes, the density (y-axis) for each plot has been normalized to a maximum value of 1.0. The high degree of overlap (similarity scores ranging from 81% to 89%) quantitatively demonstrates the exceptional fidelity of the GAN in replicating the complex distributions of the real-world data. Notably, the PDI distribution achieves the highest similarity (89%), indicating superior replication of particle size uniformity, while the particle size and zeta potential distributions maintain robust similarities of 81% and 87%, respectively. These quantitative metrics validate the GAN model's capability to generate synthetic data that closely mirrors experimental observations across multiple physicochemical dimensions.


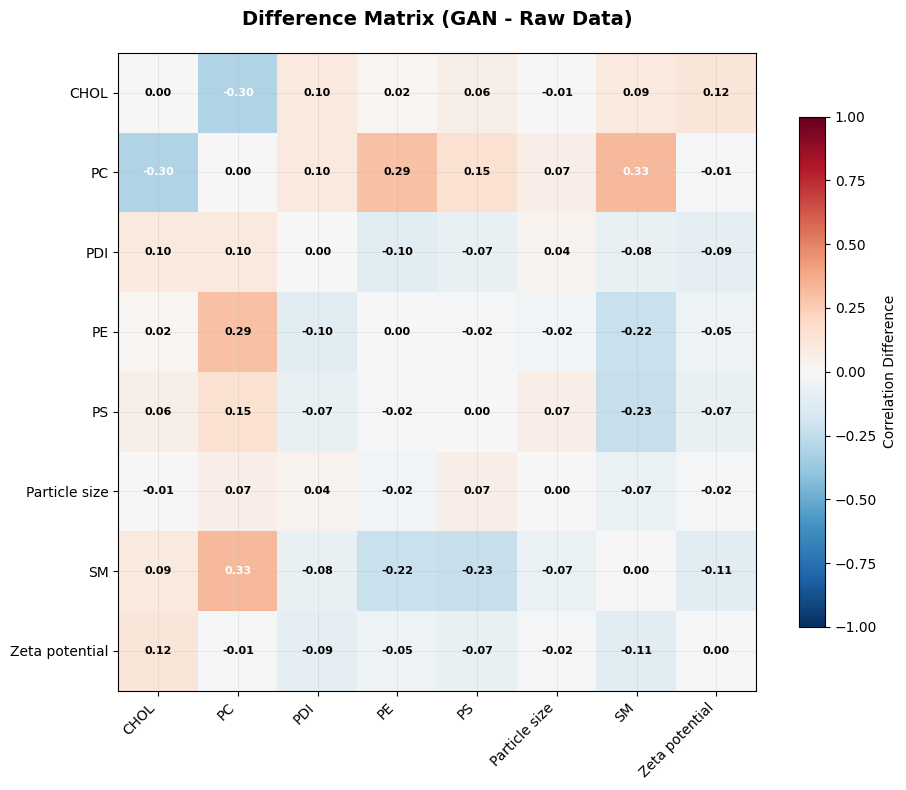
**Fig S3: Correlation difference matrix.** The matrix includes cholesterol (CHOL), phosphatidylcholine (PC), polydispersity index (PDI), phosphatidylethanolamine (PE), phosphatidylserine (PS), particle size, sphingomyelin (SM), and zeta potential. The matrix demonstrates the interdependencies present in the experimental dataset and serves as the reference baseline for evaluating the GAN model's performance. The preservation of these correlation patterns in synthetic data is quantitatively assessed through matrix correlation analysis, with the resulting coefficient indicating the degree to which inter-variable relationships are maintained in the generated dataset. The complex interdependency structure observed in this matrix establishes a challenging benchmark for evaluating the GAN model's capacity to replicate multivariate relationships in synthetic data generation.

| **Metric** | Value |
| --- | --- |
| **Matrix Correlation** | 0.8836 |
| **Mean Squared Error (MSE)** | 0.0186 |
| **Root Mean Squared Error (RMSE)** | 0.1362 |
| **Mean Absolute Error (MAE)** | 0.1040 |

**Table S1. LipidGAN model correlation matrix evaluation.** The Matrix Correlation coefficient of 0.8836 indicates preservation of inter-variable relationships in the generated dataset. The Mean Squared Error (MSE) of 0.0186 and Root Mean Squared Error (RMSE) of 0.1362 represent prediction errors when comparing individual correlation coefficients, while the Mean Absolute Error (MAE) of 0.1040 indicates that generated correlation coefficients deviate by approximately 0.10 from original values on average. These metrics demonstrate that the GAN model maintains the correlation patterns present in the training dataset while generating synthetic data with similar multivariate dependencies.

| Datasets | Critical quality attribute  (CQA) | Number  (N) | Root mean square error (RMSE) | Mean absolute error  (MAE) |
| --- | --- | --- | --- | --- |
| Experimental | Size | 225 | 0.74 | 0.53 |
|  | PDI |  | 1.01 | 0.79 |
|  | Zeta |  | 0.59 | 0.40 |
| Synthetic | Size | 17,800 | 0.23 | 0.13 |
|  | PDI |  | 0.04 | 0.01 |
|  | Zeta |  | 0.02 | 0.01 |

Table S2. Demonstrating the impact of LipidGAN-based data augmentation on the performance of hybrid algorithms.


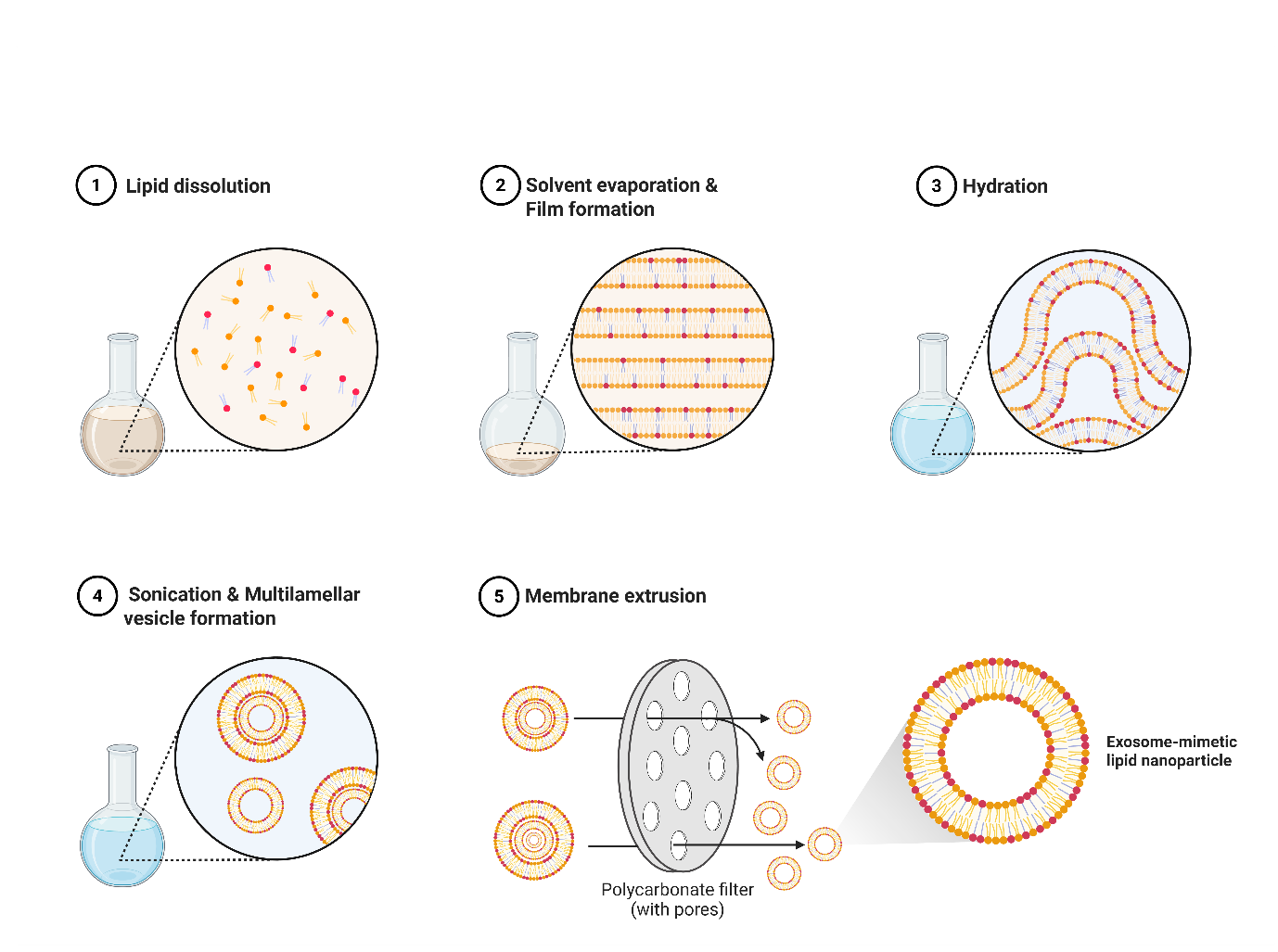


**Fig S4. Schematic illustration of the preparation process for exosome-mimetic lipid nanoparticles (ENPs).** The ENPs were synthesized using the thin-film hydration method followed by extrusion. The process involves (1-2) formation of a dried lipid film, (3-4) hydration and sonication to generate multilamellar vesicles (MLVs), and (5) sequential extrusion through polycarbonate membranes to yield uniform unilamellar vesicles (ULVs). Created in BioRender. Park, S. (2025) <https://BioRender.com/qqo2ui1>.

| Composition | % total lipid | | | | | Ave.Diameter(nm) | PDI | Zeta Potential (mV) |
| --- | --- | --- | --- | --- | --- | --- | --- | --- |
|  | CHOL | SM | PC | PS | PE |  |  |  |
| 1 | 40  -  48 | 14  -  17 | 13  -  24 | 9  -  13 | 9  -  16 | 93.5 | 0.14 | -32.04 |
| 2 |  |  |  |  |  | 142.0 | 0.14 | -33.48 |
| 3 |  |  |  |  |  | 111.9 | 0.15 | -37.54 |
| 4 |  |  |  |  |  | 97.7 | 0.19 | -36.63 |
| 5 |  |  |  |  |  | 99.8 | 0.16 | -37.85 |
| 6 |  |  |  |  |  | 112.1 | 0.13 | -31.80 |
| 7 |  |  |  |  |  | 82.9 | 0.14 | -39.67 |
| 8 |  |  |  |  |  | 92.7 | 0.15 | -34.28 |
| 9 |  |  |  |  |  | 90.3 | 0.15 | -30.48 |
| 10 |  |  |  |  |  | 98.00 | 0.16 | -37.70 |

**Table S3.** **AI-Predicted ENP Properties.** Average sizes, PDI, and zeta potentials of AI-predicted ENPs. ENP formulations were generated based on AI-predicted optimal lipid compositions using CHOL (Cholesterol), SM (Sphingomyelin), PC (Phosphatidylcholine), PS (Phosphatidylserine), and PE (Phosphatidylethanolamine). Each formulation's average particle size (nm), polydispersity index (PDI), and zeta potential (mV) were predicted by the AI model. Data are presented for 10 predicted formulations (Composition #1–#10).

| Composition | % total lipid | | | | | Ave.Diameter(nm) | PDI | Zeta Potential (mV) |
| --- | --- | --- | --- | --- | --- | --- | --- | --- |
|  | CHOL | SM | PC | PS | PE |  |  |  |
| 1 | 40  -  48 | 14  -  17 | 13  -  24 | 9  -  13 | 9  -  16 | 133.46 | 0.21 | -38.87 |
| 2 |  |  |  |  |  | 129.52 | 0.13 | -24.40 |
| 3 |  |  |  |  |  | 135.06 | 0.19 | -51.14 |
| 4 |  |  |  |  |  | 121.10 | 0.16 | -56.75 |
| 5 |  |  |  |  |  | 104.60 | 0.18 | -33.60 |
| 6 |  |  |  |  |  | 190.16 | 0.19 | -42.53 |
| 7 |  |  |  |  |  | 179.5 | 0.27 | -38.93 |
| 8 |  |  |  |  |  | 109.28 | 0.17 | -29.71 |
| 9 |  |  |  |  |  | 97.6 | 0.16 | -30.06 |
| 10 |  |  |  |  |  | 108.46 | 0.15 | -28.92 |

**Table S4: Experimental ENP Properties.** Average sizes, PDI, and zeta potentials of the ENPs with various lipid compositions. ENPs (Exosome-mimetic Nanoparticles) were formulated with different molar ratios of lipids, including CHOL (Cholesterol), SM (Sphingomyelin), PC (Phosphatidylcholine), PS (Phosphatidylserine), and PE (Phosphatidylethanolamine). Each of the 10 formulations (Composition #1–#10) was evaluated for average particle diameter (nm), polydispersity index (PDI), and zeta potential (mV) using dynamic light scattering (DLS).

|  | | Evaluation metrics | | |
| --- | --- | --- | --- | --- |
| CQAs | Ablation study models | RMSE | MAE | R^2^ |
| Particle Size | Multiple Linear regression (MLR) | 0.26 | 0.15 | 0.71 |
|  | Support Vector Machine (SVM) | 0.30 | 0.16 | 0.60 |
|  | Random Forest (RF) | 0.30 | 0.17 | 0.61 |
|  | Extreme Gradient Boosting (XGBoost) | 0.33 | 0.20 | 0.53 |
|  | Hybrid algorithm | 0.23 | 0.13 | 0.76 |
| PDI | Multiple Linear regression (MLR) | 0.05 | 0.02 | 0.22 |
|  | Support Vector Machine (SVM) | 0.05 | 0.02 | 0.25 |
|  | Random Forest (RF) | 0.05 | 0.02 | 0.25 |
|  | Extreme Gradient Boosting (XGBoost) | 0.05 | 0.03 | 0.01 |
|  | Hybrid algorithm | 0.04 | 0.01 | 0.82 |
| Zeta potential | Multiple Linear regression (MLR) | 0.03 | 0.02 | 0.87 |
|  | Support Vector Machine (SVM) | 0.05 | 0.02 | 0.58 |
|  | Random Forest (RF) | 0.04 | 0.02 | 0.69 |
|  | Extreme Gradient Boosting (XGBoost) | 0.04 | 0.03 | 0.61 |
|  | Hybrid algorithm | 0.02 | 0.01 | 0.92 |

**Table S5: Performance comparison of ENP property prediction models.** This table presents an ablation study to validate the performance of the proposed hybrid algorithm. Its ability to predict key ENP properties (Particle Size, PDI, Zeta Potential) was compared against standard models: MLR, SVM, RF, and XGBoost. The evaluation metrics—RMSE, MAE, and R²—demonstrate that the hybrid algorithm consistently provided the most accurate predictions, confirming its selection for this study.


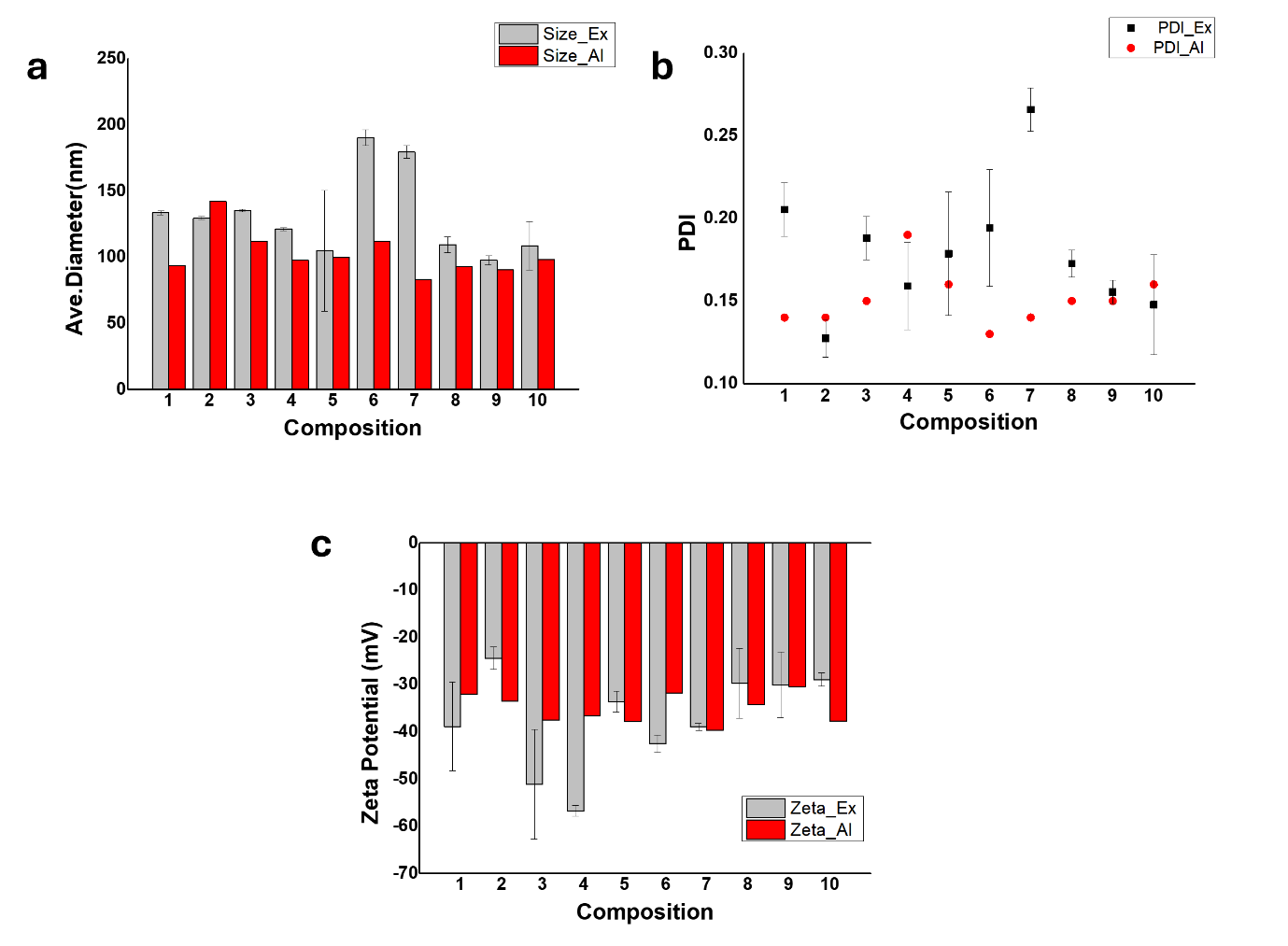


**Fig S5: AI vs. Experimental Property Comparison. (a)** Particle sizes of exosome-mimetic lipid nanoparticles measured experimentally (gray) and predicted by the AI model (red). **(b)** Polydispersity index (PDI) values determined experimentally (black squares) and predicted by AI (red circles). **(c)** Zeta potentials obtained through experimental measurements (gray) and AI-based predictions (red). Across all lipid compositions (#1–#13), the AI model demonstrates strong agreement with experimental outcomes, particularly in size and zeta potential predictions. These results validate the predictive performance of the AI-assisted formulation model for designing stable, well-dispersed exosome-mimetic nanoparticles.

| Composition | Control | 10⁶ | 10⁷ | 10⁸ | 10⁹ | 10¹⁰ |
| --- | --- | --- | --- | --- | --- | --- |
| 1 | 100.00±5.19 | 138.52±15.83 | 140.24±18.34 | 133.65±17.04 | 130.39±17.82 | 121.67±13.78 |
| 2 | 100.00±5.43 | 115.54±24.43 | 117.05±18.07 | 124.99±17.92 | 119.20±8.17 | 117.72±13.93 |
| 3 | 100.00±6.65 | 108.77±13.55 | 106.63±11.02 | 110.41±9.39 | 104.18±14.98 | 97.83±11.05 |
| 4 | 100.00±8.77 | 105.63±3.34 | 106.12±3.99 | 109.70±6.14 | 110.66±12.31 | 101.38±4.56 |
| 5 | 100.00±9.90 | 113.34±11.86 | 115.48±11.92 | 111.35±11.85 | 119.69±4.68 | 114.64±7.13 |
| 6 | 100.00±1.41 | 105.10±3.98 | 106.22±3.68 | 109.80±4.62 | 107.06±2.12 | 107.85±2.92 |
| 7 | 100.00±15.16 | 111.55±16.01 | 115.83±12.57 | 115.15±12.05 | 113.66±10.94 | 112.13±9.06 |
| 8 | 100.00±6.29 | 119.21±8.69 | 118.83±9.35 | 115.17±12.85 | 109.36±5.50 | 105.81±5.85 |
| 9 | 100.00±9.97 | 110.05±8.52 | 109.79±5.61 | 109.43±9.15 | 111.76±8.48 | 107.42±8.96 |
| 10 | 100.00±5.37 | 111.93±10.11 | 113.01±9.06 | 109.00±5.78 | 107.79±5.89 | 105.25±4.12 |

**Table S6.** **HeLa Cell Viability.** This table shows the comparative viability of HeLa cells (%) after treatment with different EXOAI compositions. HeLa cells were treated with exosome-mimetic lipid nanoparticles formulated with 10 different lipid compositions (#1–#10) at increasing particle concentrations ranging from 10⁶ to 10¹⁰ particles per well. Cell viability was assessed using a WST-based assay after 24 hours of incubation. Data are presented as mean ± standard deviation (n = 5). Control represents untreated cells, normalized to 100%. No significant cytotoxicity was observed in most compositions across tested concentrations, with viability values remaining above 90% in the majority of conditions. Minor reductions were observed at the highest dose (10¹⁰ particles/well) for compositions #3 and #6.

| Composition | Control | 10⁶ | 10⁷ | 10⁸ | 10⁹ | 10¹⁰ |
| --- | --- | --- | --- | --- | --- | --- |
| 1 | 100.00±5.16 | 104.82±5.24 | 105.77±7.61 | 105.13±8.38 | 106.75±9.07 | 105.31±5.44 |
| 2 | 100.00±7.45 | 96.88±10.22 | 97.74±6.81 | 91.72±6.80 | 91.66±7.23 | 89.86±6.66 |
| 3 | 100.00±2.25 | 105.36±6.03 | 109.17±6.72 | 106.33±8.51 | 106.35±8.44 | 106.10±11.75 |
| 4 | 100.00±6.94 | 96.50±6.06 | 93.78±4.29 | 92.08±1.88 | 96.53±4.68 | 95.60±3.70 |
| 5 | 100.00±4.53 | 100.54±8.93 | 103.39±7.13 | 103.90±8.06 | 103.59±6.91 | 107.55±9.68 |
| 6 | 100.00±6.14 | 99.09±6.53 | 98.77±8.40 | 96.16±5.40 | 98.39±6.41 | 99.50±6.95 |
| 7 | 100.00±4.89 | 105.22±6.74 | 104.06±6.59 | 108.14±5.21 | 110.70±4.13 | 106.63±4.00 |
| 8 | 100.00±2.62 | 107.64±10.20 | 107.19±6.17 | 106.31±5.56 | 101.19±9.33 | 98.28±6.10 |
| 9 | 100.00±2.51 | 104.84±4.22 | 103.93±7.60 | 106.41±5.68 | 100.08±3.67 | 103.60±5.38 |
| 10 | 100.00±7.82 | 103.34±5.30 | 99.12±11.07 | 101.55±5.06 | 100.49±5.19 | 99.80±2.18 |

**Table S7**. **MCF-7 Cell Viability.** This table details the comparative viability of MCF7 cells (%) after treatment with different EXOAI compositions. MCF-7 cells were treated with exosome-mimetic lipid nanoparticles formulated using 10 different lipid compositions (#1–#10) across increasing particle concentrations (10⁶ to 10¹⁰ particles per well). Cell viability was measured using a WST-based assay after 24 hours of incubation. Data are shown as mean ± standard deviation (n = 5). The untreated control condition was normalized to 100%. Most compositions showed high viability (>90%) across the full concentration range, indicating low cytotoxicity. Slight decreases in viability were observed at the highest concentrations (10¹⁰ particles/well) in a few formulations (e.g., #2, #4, #8), suggesting mild dose-dependent effects in some cases.

| Composition | Control | 10⁶ | 10⁷ | 10⁸ | 10⁹ | 10¹⁰ |
| --- | --- | --- | --- | --- | --- | --- |
| 1 | 100.00±4.99 | 102.43±2.70 | 103.87±4.06 | 100.98±2.72 | 101.90±6.43 | 99.74±3.83 |
| 2 | 100.00±1.37 | 100.29±5.35 | 101.47±3.25 | 102.28±2.48 | 101.41±1.03 | 101.68±1.47 |
| 3 | 100.00±4.85 | 105.25±5.04 | 102.41±3.28 | 103.60±1.80 | 103.25±1.02 | 107.32±3.34 |
| 4 | 100.00±5.35 | 96.25±0.59 | 102.08±1.82 | 101.59±1.84 | 97.85±4.08 | 96.58±6.36 |
| 5 | 100.00±7.49 | 106.68±1.58 | 106.72±5.41 | 108.71±2.19 | 103.24±4.11 | 106.00±2.62 |
| 6 | 100.00±5.18 | 96.19±2.43 | 96.76±3.48 | 93.73±2.70 | 98.14±2.67 | 90.73±4.03 |
| S7 | 100.00±1.72 | 98.86±2.99 | 100.89±3.37 | 102.40±6.02 | 101.31±1.38 | 100.69±2.29 |
| 8 | 100.00±6.23 | 94.96±3.01 | 104.69±3.14 | 104.77±1.36 | 100.01±6.03 | 92.64±4.63 |
| 9 | 100.00±6.40 | 106.85±4.69 | 99.89±3.12 | 104.16±2.87 | 99.99±1.28 | 103.69±1.13 |
| 10 | 100.00±3.62 | 104.22±5.19 | 96.68±3.16 | 103.83±2.69 | 93.65±3.12 | 106.07±6.03 |

**Tables S8.** **H1975 Cell Viability.** This table presents the comparative viability of H1975 cells (%) after treatment with different EXOAI compositions. H1975 cells were treated with exosome-mimetic lipid nanoparticles formulated with 10 different compositions (#1–#10) at increasing concentrations ranging from 10⁶ to 10¹⁰ particles per well. Cell viability was assessed using a WST-based assay after 24 hours of treatment. Data are presented as mean ± standard deviation (n = 5), with untreated control values normalized to 100%. Most formulations showed minimal cytotoxicity, maintaining viability above 90% across all tested concentrations. Composition #8 displayed slightly reduced viability at higher concentrations (≥10⁹ particles/well), indicating possible mild concentration-dependent effects in H1975 cells.

| Composition | 0 day | | | 3 day | | | | | 7 day | | | | |  |  |  |
| --- | --- | --- | --- | --- | --- | --- | --- | --- | --- | --- | --- | --- | --- | --- | --- | --- |
|  | Ave.Diameter  (nm) | | P.I. | | | Ave.Diameter  (nm) | | P.I. | | | Ave.Diameter  (nm) | | P.I. | | | |
| 1 | 133.50±0.60 | | 0.21±0.01 | | | 108.13±0.28 | | 0.21±0.02 | | | 104.34±1.12 | | 0.20±0.02 | | | |
| 2 | 129.40±1.00 | | 0.13±0.01 | | | 105.06±0.77 | | 0.14±0.02 | | | 110.08±0.72 | | 0.13±0.02 | | | |
| 3 | 134.77±1.05 | | 0.19±0.01 | | | 110.60±1.11 | | 0.19±0.01 | | | 107.82±0.41 | | 0.19±0.01 | | | |
| 4 | 121.03±1.10 | | 0.16±0.02 | | | 94.96±0.59 | | 0.18±0.01 | | | 91.38±0.37 | | 0.16±0.01 | | | |
| 5 | 107.90±0.89 | | 0.18±0.01 | | | 129.08±1.53 | | 0.16±0.02 | | | 98.6±3.08 | | 0.16±0.03 | | | |
| 6 | 190.46±17.90 | | 0.19±0.05 | | | 300.34±59.94 | | 0.23±0.03 | | | 222.64±16.07 | | 0.18±0.02 | | | |
| 7 | 178.67±3.81 | | 0.27±0.02 | | | 223.66±18.50 | | 0.20±0.01 | | | 221.24±21.98 | | 0.19±0.01 | | | |
| 8 | 108.00±3.36 | | 0.17±0.01 | | | 102.30±0.65 | | 0.17±0.02 | | | 101.04±1.67 | | 0.18±0.00 | | | |
| 9 | 96.60±1.61 | | 0.16±0.01 | | | 93.38±0.96 | | 0.16±0.01 | | | 91.82±1.13 | | 0.18±0.02 | | | |
| 10 | 101.53±4.91 | | 0.15±0.02 | | | 98.52±1.93 | | 0.16±0.01 | | | 96.94±1.81 | | 0.18±0.04 | | | |
| Composition | 10 day | | | 13 day | | | | | 17 day | | | | | |  |  |
|  | Ave.Diameter(nm) | P.I. | | | Ave.Diameter(nm) | | P.I. | | | Ave.Diameter(nm) | | P.I. | | | |  |
| 1 | 105.26±0.96 | 0.18±0.01 | | | 103.96±2.29 | | 0.20±0.01 | | | 105.33±0.06 | | 0.19±0.00 | | | |  |
| 2 | 98.67±1.00 | 0.14±0.01 | | | 98.73±1.32 | | 0.15±0.03 | | | 99.43±0.47 | | 0.13±0.00 | | | |  |
| 3 | 107.80±0.75 | 0.19±0.00 | | | 107.80±1.90 | | 0.19±0.01 | | | 104.90±0.66 | | 0.18±0.01 | | | |  |
| 4 | 91.30±0.26 | 0.16±0.00 | | | 90.00±0.56 | | 0.17±0.02 | | | 91.06±0.67 | | 0.17±0.01 | | | |  |
| 5 | 119.83±19.93 | 0.15±0.07 | | | 132.10±36.00 | | 0.15±0.03 | | | 140.00±17.04 | | 0.16±0.04 | | | |  |
| 6 | 215.13±20.48 | 0.16±0.01 | | | 234.16±2.55 | | 0.16±0.00 | | | 240.83±12.41 | | 0.16±0.01 | | | |  |
| 7 | 214.96±15.21 | 0.18±0.03 | | | 218.16±7.02 | | 0.15±0.01 | | | 216.76±4.44 | | 0.16±0.02 | | | |  |
| 8 | 101.20±0.44 | 0.18±0.03 | | | 101.60±1.15 | | 0.15±0.02 | | | 91.33±0.46 | | 0.16±0.02 | | | |  |
| 9 | 91.67±0.21 | 0.17±0.02 | | | 92.00±0.60 | | 0.18±0.01 | | | 93.16±0.40 | | 0.15±0.01 | | | |  |
| 10 | 96.83±1.12 | 0.17±0.01 | | | 96.53±0.92 | | 0.16±0.01 | | | 97.46±2.02 | | 0.17±0.02 | | | |  |

**Table S9. ENP Stability Over Time.** ENP Stability Over Time. The average particle diameter (nm) and polydispersity index (PDI) of exosome-mimetic lipid nanoparticles (ENPs) were monitored over 17 days to assess colloidal stability. Measurements were taken at 0, 3, 7, 10, 13, and 17 days post-synthesis using dynamic light scattering (DLS). Most formulations (#1–#5, #8–#10) maintained consistent size and PDI values over time, indicating good physical stability.


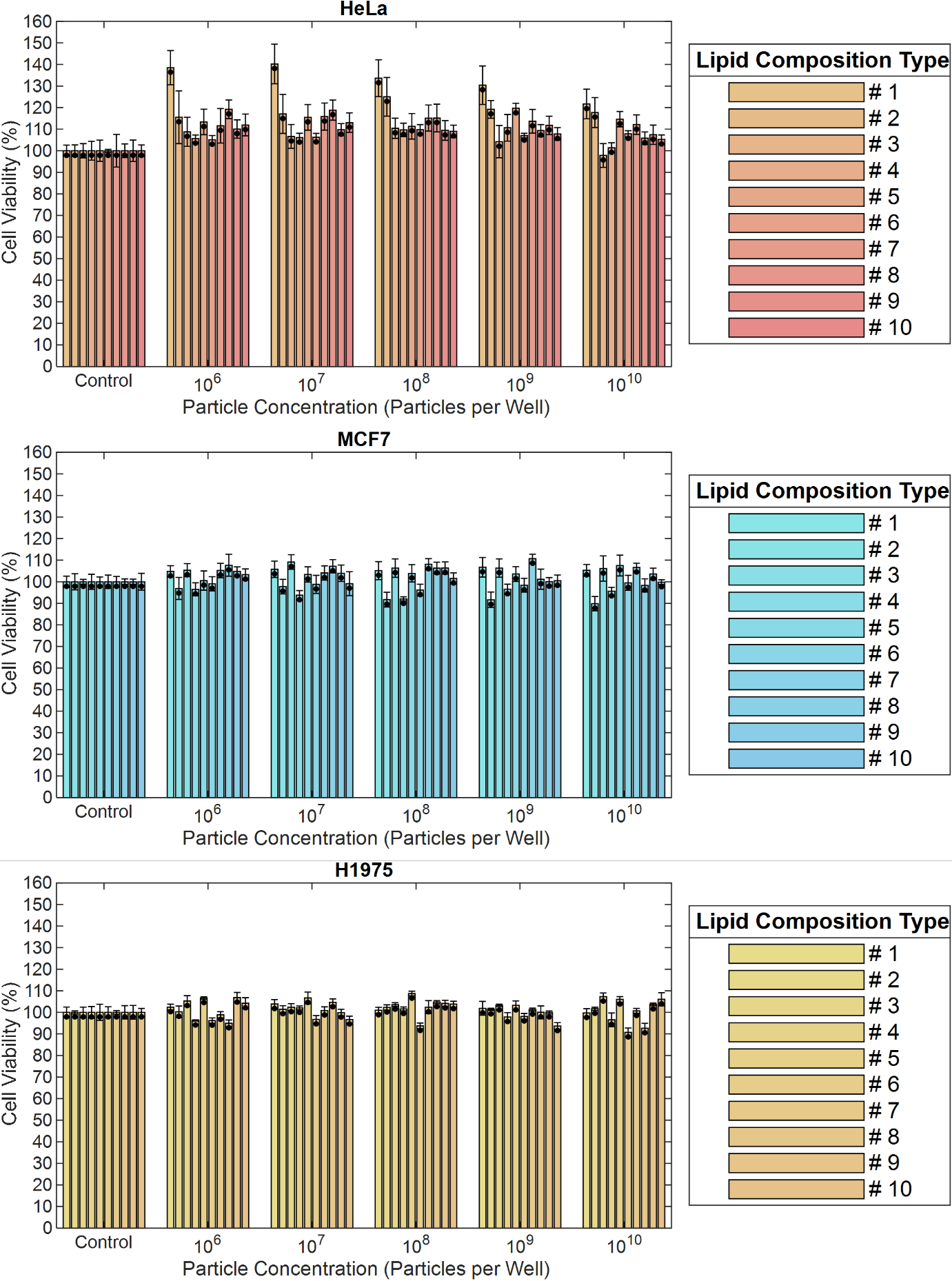


**Fig S6: Cell Viability Summary.** This figure summarizes the viability of HeLa, MCF-7, and H1975 cells after treatment with Rhodamine-labeled exosome-mimetic lipid nanoparticles. Cell viability was assessed in HeLa, MCF-7, and H1975 cells following treatment with exosome-mimetic lipid nanoparticles of varying lipid compositions (#1–#10) across a range of particle concentrations (10⁶ to 10¹⁰ particles per well). Control groups (untreated) were included for each cell line. All three cell lines maintained high viability (>90%) across most conditions, indicating low cytotoxicity of the nanoparticles. These results confirm that the exosome-mimetic nanoparticles were well tolerated by the cells and suggest successful cellular internalization without significant toxicity. The consistency of cell viability across increasing particle doses supports the biocompatibility of the designed lipid formulations.


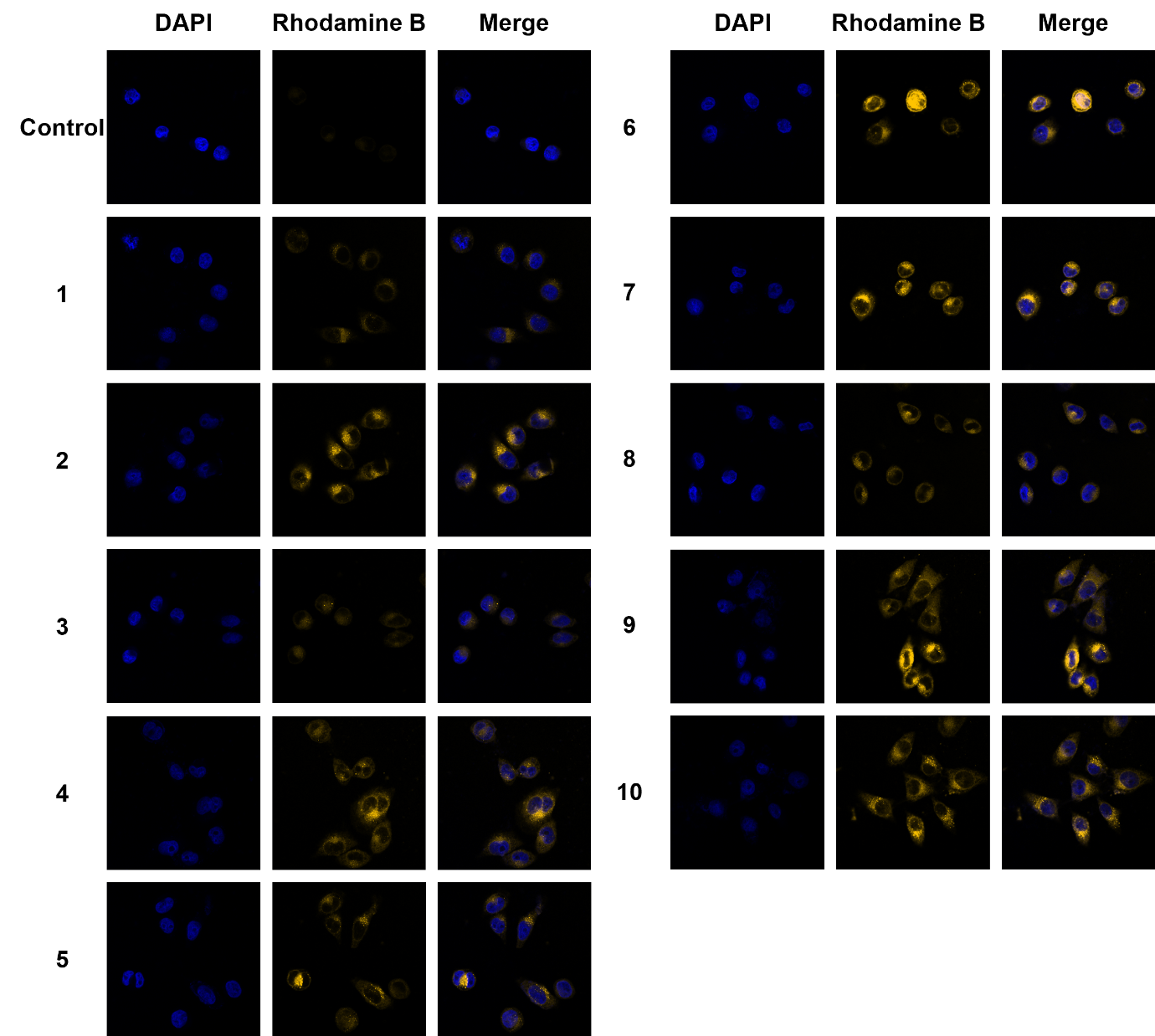


**Fig S7: HeLa Confocal Imaging.** This figure displays confocal microscopy images of Rhodamine B-labeled nanoparticles internalized by HeLa cells, taken at 40× magnification. Confocal fluorescence microscopy images of HeLa cells incubated with Rhodamine B-labeled nanoparticles of 10 different lipid compositions (#1–#10). Cells were visualized at 40× magnification. Nuclei were stained with DAPI (blue), and nanoparticles were visualized via Rhodamine B fluorescence (yellow). The images confirm intracellular localization of nanoparticles, indicating successful cellular uptake in most treatment conditions. Differences in uptake efficiency among the formulations were clearly observed, reflecting the influence of lipid composition on cellular internalization.


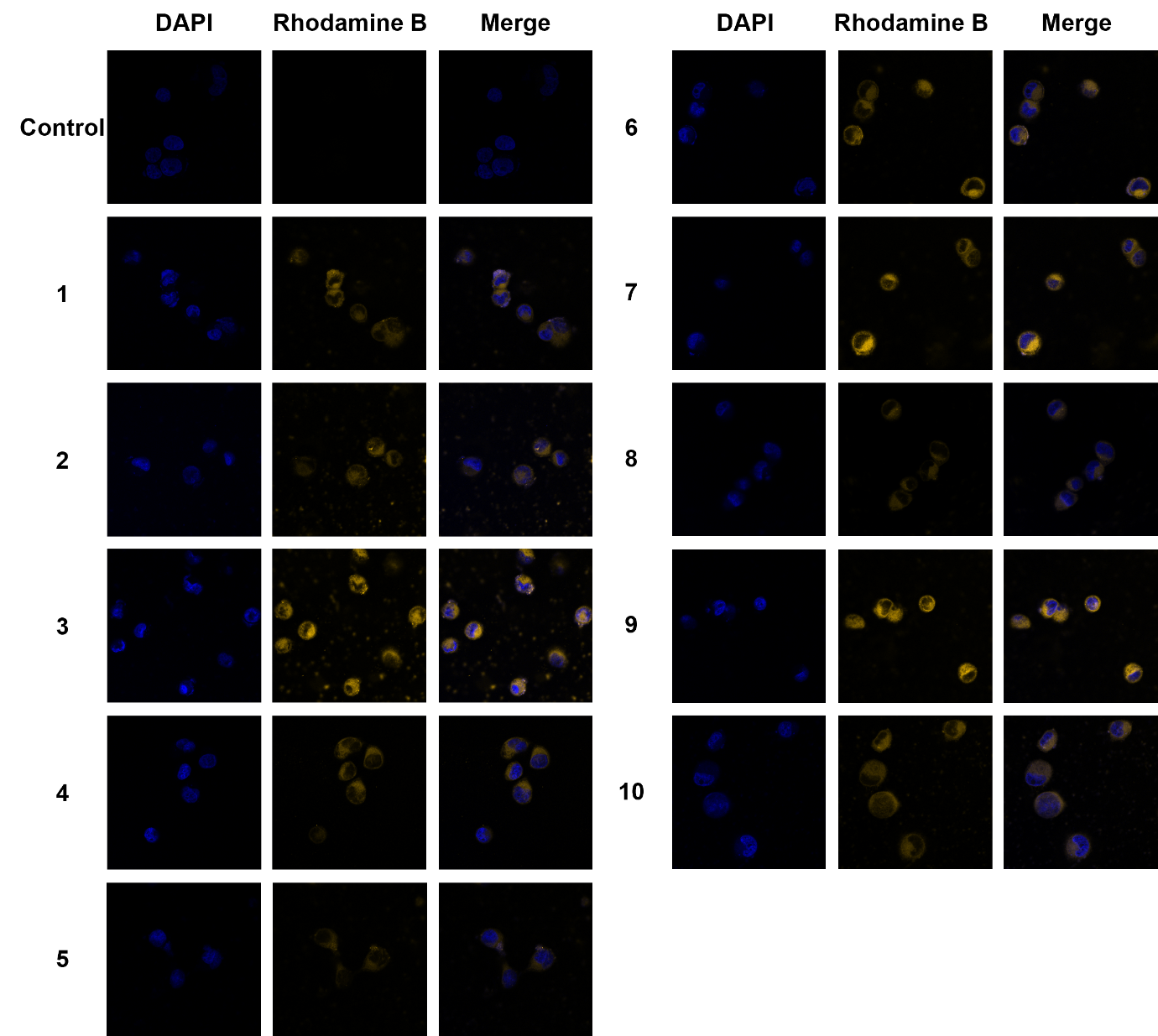


**Fig S8: MCF-7 Confocal Imaging.** This figure presents confocal microscopy images of Rhodamine B-labeled nanoparticles internalized by MCF-7 cells at 40× magnification. The images feature MCF-7 cells incubated with nanoparticles from 10 different lipid compositions (#1–#10). Nuclei were stained with DAPI (blue), and nanoparticles were visualized via Rhodamine B fluorescence (yellow). The images confirm intracellular localization of nanoparticles, indicating successful cellular uptake in most treatment conditions. Differences in uptake efficiency among the formulations were clearly observed, reflecting the influence of lipid composition on cellular internalization.


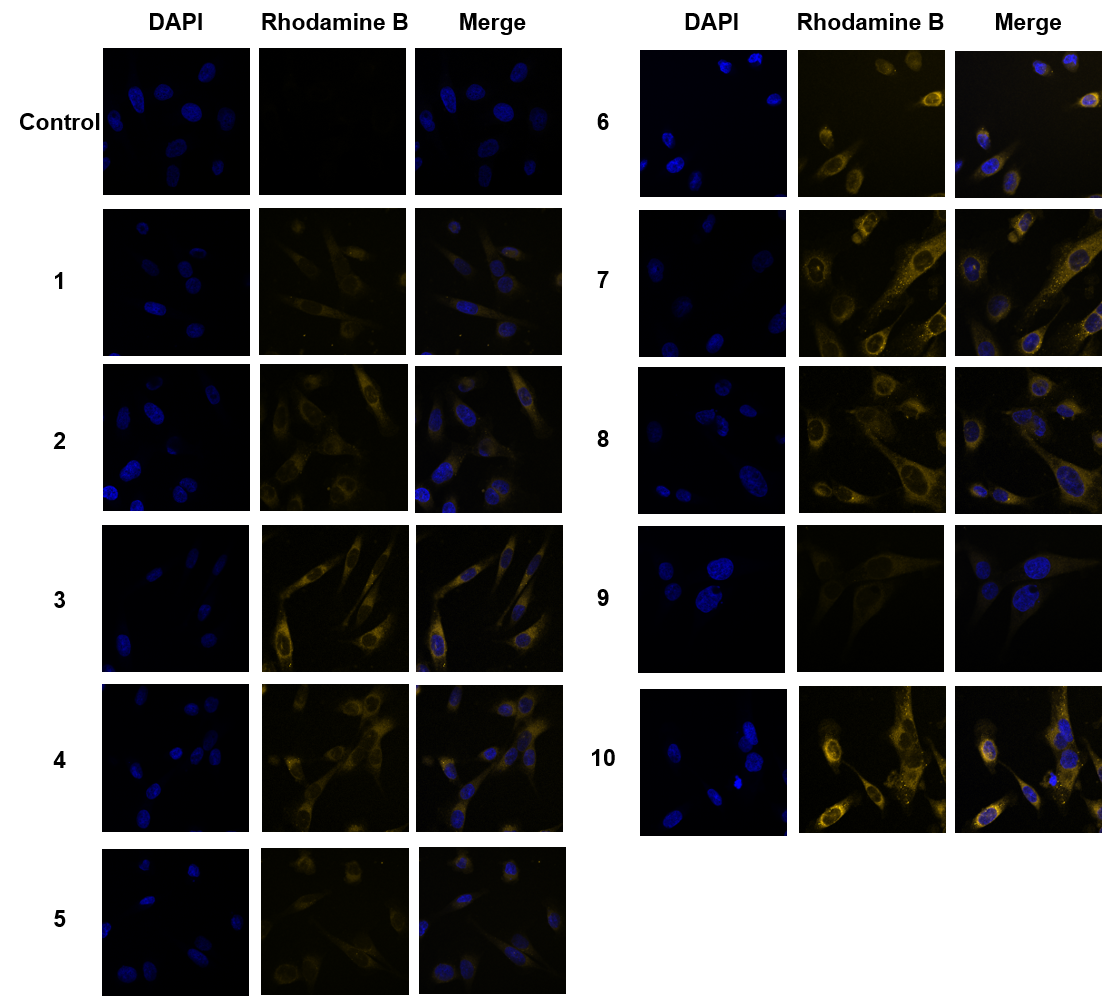


**Fig S9: H1975 Confocal Imaging.** This figure contains confocal fluorescence microscopy images of H1975 cells incubated with Rhodamine B-labeled nanoparticles. The cells, visualized at 40x magnification, correspond to 10 different lipid compositions (#1–#10). Nuclei were stained with DAPI (blue), and nanoparticles were visualized via Rhodamine B fluorescence (yellow). Images correspond to 10 different lipid compositions (#1–#10). The images confirm intracellular localization of nanoparticles, indicating successful cellular uptake in most treatment conditions. Differences in uptake efficiency among the formulations were clearly observed, reflecting the influence of lipid composition on cellular internalization.


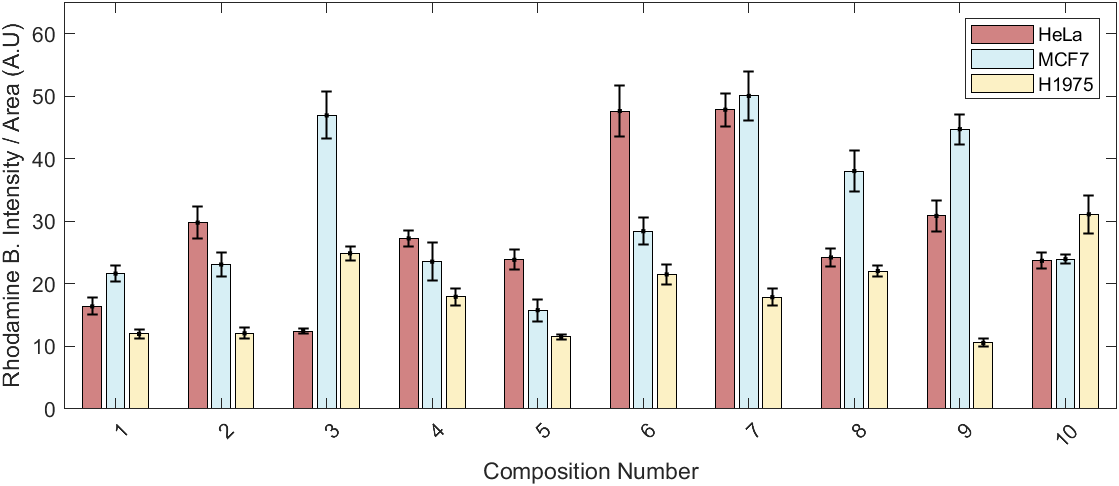


**Fig S10: Fluorescence vs. Bright-Field Imaging**. This figure provides a quantitative analysis of Rhodamine B fluorescence intensity per unit cell area in HeLa, MCF-7, and H1975 cells after incubation with exosome-mimetic lipid nanoparticles. Fluorescence intensities were obtained from the internalized nanoparticles for each of the 10 lipid compositions (#1–#10) to compare cellular uptake. For each condition, images from three independent microscopic fields were analyzed to ensure reproducibility. Intensities were background-subtracted, normalized to the total cell area in each image, and expressed as arbitrary units (A.U.). Data are presented as standard error of the mean (n = 3 fields). Higher fluorescence intensity indicates greater cellular uptake of nanoparticles. MCF-7 and HeLa cells exhibited composition-dependent variations in uptake, with certain formulations (e.g., #3, #6, #7, #9) showing markedly higher intensities, while H1975 cells consistently displayed lower uptake across all compositions.

**
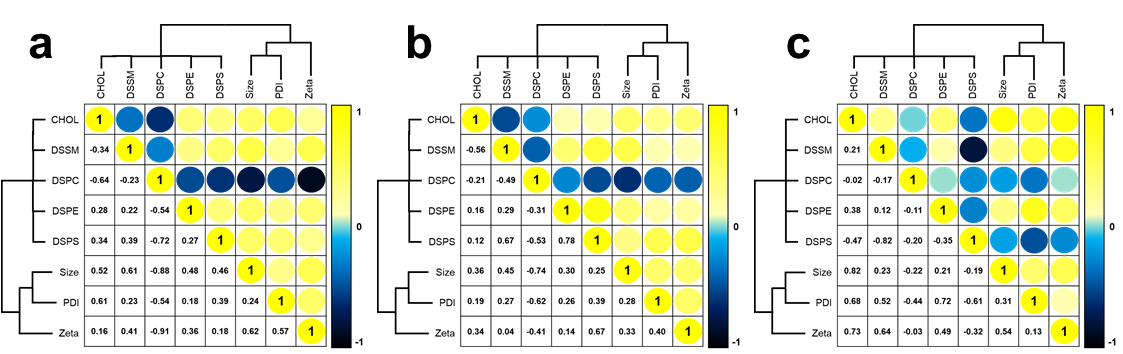
**

**Fig S11: Correlation matrix results for the three cancer cell lines.**
(a) Correlation matrix for the HeLa cell line (positive correlations shown in yellow, negative correlations in blue). (b) Correlation matrix for the MCF-7 cell line. (c) Correlation matrix for the H1975 cell line. Notably, the H1975 cell line exhibits a negative correlation in the PC and PS classes.


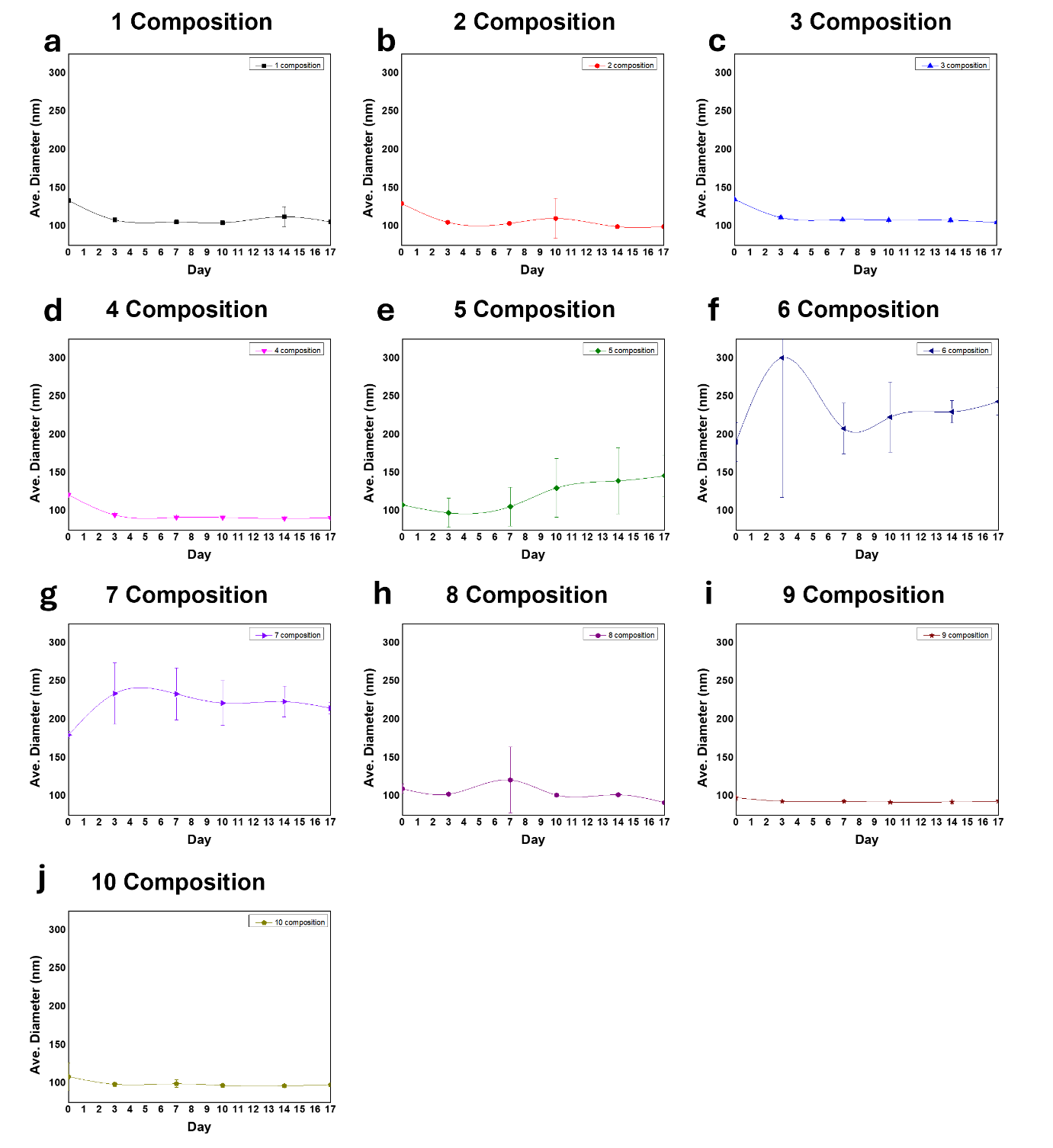


**Fig S12: Size Stability of ENPs.**
(a–j) Time-dependent changes in the polydispersity index (PDI) of exosome-mimetic lipid nanoparticles (ENPs) with lipid compositions #1–#10, monitored over the evaluation period using dynamic light scattering (DLS). Individual plots are presented as mean ± SD from five independent measurements (n = 5), indicating that most formulations maintained PDI values below 0.3 during storage.


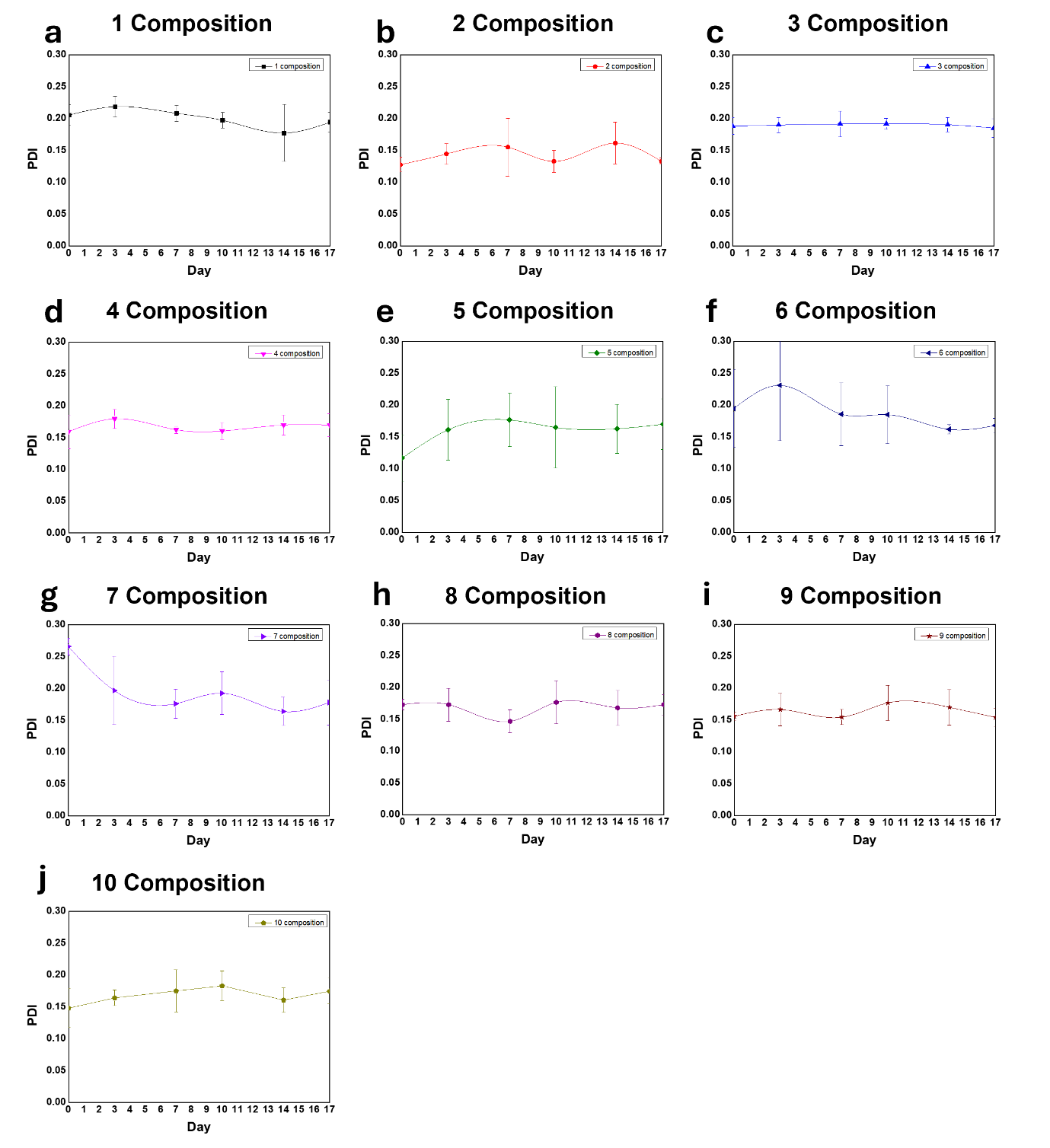


**Fig S13: PDI Stability of ENPs.**(a–j) Time-dependent changes in the polydispersity index (PDI) of exosome-mimetic lipid nanoparticles (ENPs) with lipid compositions #1–#10, monitored over the evaluation period using dynamic light scattering (DLS). Individual plots are presented as mean ± SD from five independent measurements (n = 5), indicating that most formulations maintained PDI values below 0.3 during storage.


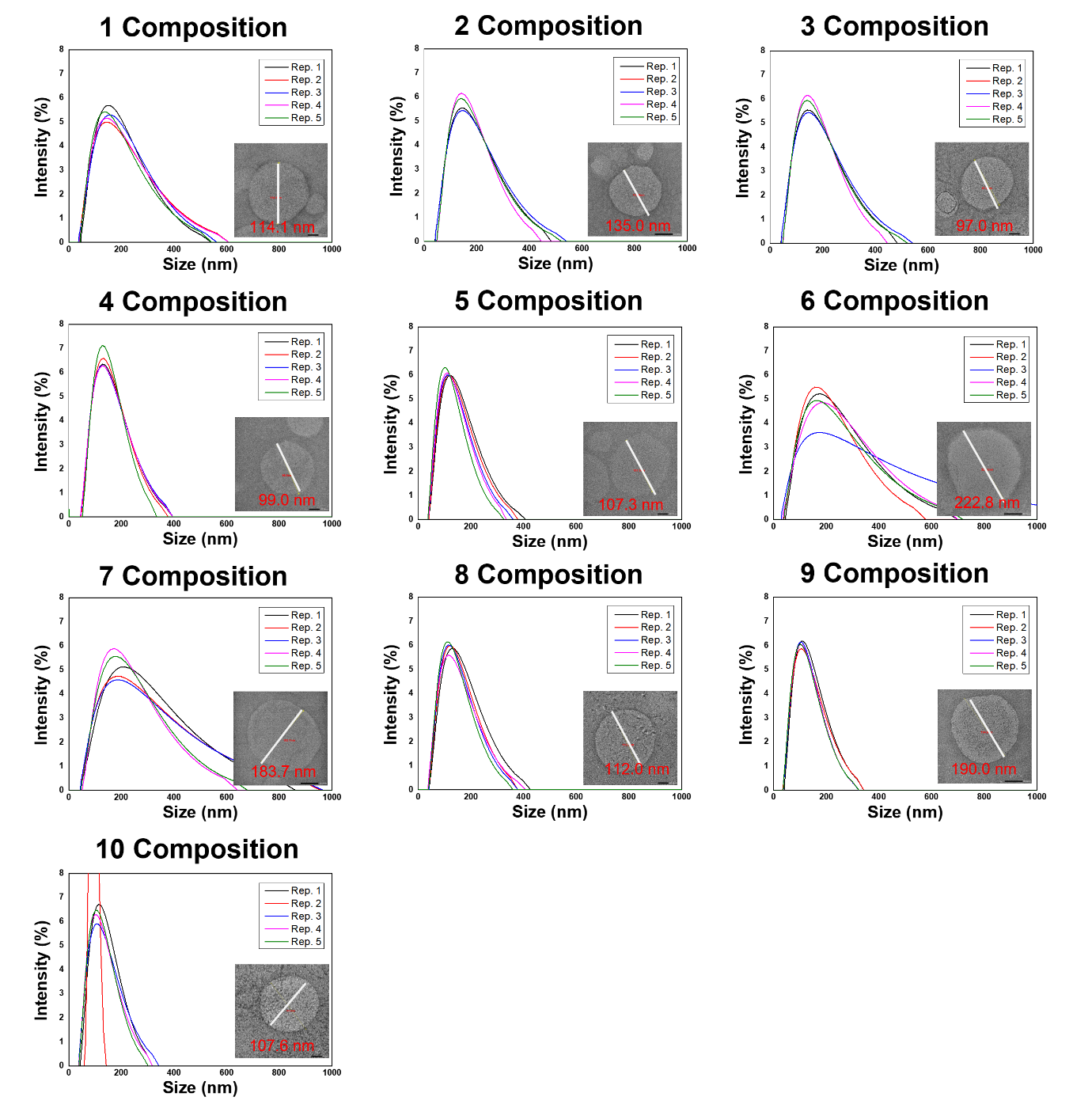


**Fig S14:** **DLS & TEM of ENP Compositions.** Dynamic light scattering (DLS) and transmission electron microscopy (TEM) analysis of exosome-mimetic lipid nanoparticles with different lipid compositions. DLS intensity distributions of exosome-mimetic lipid nanoparticles with lipid compositions #1–#10. Each graph shows the size distribution profiles obtained from five independent replicates (Rep 1–5), confirming reproducibility and monodispersity of most formulations. The accompanying TEM images for each composition provide morphological validation and confirm particle sizes consistent with DLS measurements. Observed nanoparticle sizes ranged from approximately 90 nm to 220 nm depending on lipid composition, with round morphology and well-defined boundaries. Together, these data validate the formation of nanoscale vesicles with composition-dependent size characteristics.
